# The FKBPL-based therapeutic peptide, AD-01, protects the endothelium from hypoxia-induced damage by stabilising hypoxia inducible factor-α and inflammation

**DOI:** 10.1186/s12967-025-06118-w

**Published:** 2025-03-11

**Authors:** Sahar Ghorbanpour, Siân Peta Cartland, Hao Chen, Sanchit Seth, Rupert C. Ecker, Claire Richards, Dunja Aksentijevic, Matthew P. Padula, Louise Cole, Majid Ebrahimi Warkiani, Mary Meltem Kavurma, Lana McClements

**Affiliations:** 1 School of Life Sciences & Institute for Biomedical Materials and Devices, Faculty of Science, University of Technology Sydney, Sydney, NSW Australia; 2 Heart Research Institute, The University of Sydney, Sydney, NSW Australia; 3 School of Biomedical Sciences, Faculty of Health, Queensland University of Technology, Brisbane, QLD Australia; 4 TissueGnostics Australia Pty Ltd, Brisbane, Australia; 5 Translational Research Institute, 37 Kent Street, Woolloongabba, QLD 4102 Australia; 6 Centre for Biochemical Pharmacology, School of Medicine and Dentistry, William Harvey Research Institute, Barts and the London, Queen Mary University of London, London, UK; 7 The Australian Institute for Microbiology and Infection (AIMI), Faculty of Science, University of Technology Sydney, Sydney, NSW Australia; 8 School of Biomedical Engineering, Faculty of Engineering and Information Technology, University of Technology Sydney, Sydney, NSW Australia

**Keywords:** FKBPL, Angiogenesis, Hypoxia, Inflammation, Endothelial dysfunction, Microfluidics

## Abstract

**Background:**

Endothelial dysfunction is a hallmark feature of cardiovascular disease (CVD), yet the underlying mechanisms are still poorly understood. This has impeded the development of effective therapies, particularly for peripheral artery disease. FK506-binding protein like (FKBPL) and its therapeutic peptide mimetic, AD-01, are crucial negative regulators of angiogenesis, however their roles in CVD are unknown. In this study, we aimed to elucidate the FKBPL-mediated mechanisms involved in regulating endothelial dysfunction induced by hypoxia or inflammation, and to determine whether AD-01 can effectively restore endothelial function under these conditions.

**Methods:**

Hindlimb ischemia was induced in mice by ligating the proximal and distal ends of the right femoral artery, and, after three days, the gastrocnemius muscle was collected for immunofluorescence staining, and RNA extraction. A 3D in vitro microfluidics model was developed to determine the endothelial cell migration and impact of FKBPL following treatments with: (i) 24 µM FKBPL targeted siRNA, (ii) 1 mM hypoxia inducible factor (HIF-1)α activator (DMOG), (iii) 50% (v/v) macrophage conditioned media (MCM), ± 100 nM AD-01. Unbiased, untargeted proteomic analysis was conducted via LC-MS/MS to identify protein targets of AD-01.

**Results:**

FKBPL expression is substantially downregulated in mice after hindlimb ischemia (*p* < 0.05, protein; *p* < 0.001, mRNA), correlating with increased neovascularization and altered vascular adhesion molecule expression. In our real-time advanced 3D microfluidics model, hypoxia suppressed FKBPL (*p* < 0.05) and VE-cadherin (*p* < 0.001) expression, leading to increased endothelial cell number and migration (*p* < 0.001), which was restored by AD-01 treatment (*p* < 0.01). Under inflammatory conditions, FKBPL (*p* < 0.01) and HIF-1α (*p* < 0.05) expression was elevated, correlating with increased endothelial cell migration (*p* < 0.05). Unlike hypoxia, AD-01 did not influence endothelial cell migration under inflammatory conditions, but normalized FKBPL (*p* < 0.001), HIF-1α (*p* < 0.05) and CD31 (*P* < 0.05), expression, in 3D microfluidic cell culture. Proteomic analysis revealed that AD-01 treatment in hypoxia enhanced the abundance of tissue remodelling and vascular integrity proteins including collagen alpha-1(XIX) chain and junctional cadherin associated-5 (JCAD) proteins.

**Conclusions:**

FKBPL represents an important novel mechanism in hypoxia and inflammation-induced angiogenesis. The FKBPL-based therapeutic peptide, AD-01, could be a viable treatment option for CVD-related endothelial cell dysfunction.

**Supplementary Information:**

The online version contains supplementary material available at 10.1186/s12967-025-06118-w.

## Introduction

Cardiovascular disease (CVD) is the leading cause of death worldwide, and its hallmark feature is endothelial cell dysfunction [1]. Endothelial cells, the inner layer of blood vessels, are responsible for maintaining haemostasis, fibrinolysis, regulating vascular tone and permeability, and mediating the inflammatory response [2–5]. Although hypoxia and inflammation are critical drivers of endothelial cell dysfunction and maladaptive vascular responses in CVD [6], the underlying mechanisms contributing to endothelial dysfunction leading to CVD remain poorly understood. Greater understanding could pave the way for the development of more effective therapeutics aimed at improving endothelial cell function(s).

Angiogenesis is a multistep process that is initiated by the activation, proliferation, and migration of endothelial cells, leading to endothelial cell sprouting through the extracellular matrix (ECM) [7]. Endothelial cell dysfunction can lead to aberrant angiogenesis, which is characterised by angiogenic imbalance affecting normal physiological vascular homeostasis. Some of the key factors involved in this process include hypoxia inducible factor (HIF)-1α, vascular endothelial growth factor (VEGF), fibroblast growth factor (FGF)-1, FGF-2, and tumour necrosis factor (TNF)-α [8–11].

FK506-binding protein like (FKBPL) is a critical factor in physiological and pathological angiogenesis and is a divergent member of the immunophilin protein family. FKBPL is expressed and secreted predominantly by fibroblasts and endothelial cells. Interestingly, FKBPL homozygous knockout mice, *fkbpl*^*−/−*^ are embryonic lethal, whereas *fkbpl*^*+/−*^ heterozygous knockdown mice develop normally albeit with early signs of endothelial and vascular dysfunction [12]. The amino acid 34 to 57 regions on the N-terminus of FKBPL protein, outside of the HSP90 binding region, are potent angiogenesis inhibitors of tumour and endothelial cell migration [12,13]. Several studies have demonstrated the important anti-cancer role of FKBPL and its therapeutic peptide derivatives, AD-01 (a preclinical peptide candidate) and ALM201 (clinical peptide candidate), which target angiogenesis via CD44^14,15^. Unlike other anti-angiogenic agents, FKBPL-based peptides are not cytotoxic to cells and have shown good safety in both mice and humans [14,15]. Additionally, AD-01 acts both as a vascular stabilizer and an anti-inflammatory agent under pro-inflammatory conditions by modulating the signalling pathways of VE-cadherin and nuclear factor kappa-light chain enhancer of activated B cells (NF-κB) [16].

Our study used an integrated experimental approach incorporating in vivo (hindlimb ischemia mouse model) and ex vivo (3D microfluidic microvascular endothelial cell model) systems to demonstrate, at the single cell level, that FKBPL plays a pivotal role in hypoxia and inflammation-induced endothelial cell dysfunction. This is the first study to evaluate the therapeutic potential of AD-01 in addressing hypoxia-induced endothelial cell effects using an advanced real-time 3D microfluidics model. To fully elucidate FKBPL-mediated endothelial cell mechanisms, we performed untargeted and unbiased proteomics analysis and identified key proteins and pathways in hypoxic settings relevant to the early pathogenesis of CVD. Collectively, these data suggest that FKBPL plays a critical role in regulating HIF-1α signalling under hypoxic and inflammatory conditions in endothelial cells. AD-01 represents a novel and targeted therapeutic candidate for CVD that is capable of abrogating the dysregulated FKBPL-HIF-1α mechanism and inflammation, hence abrogating endothelial cell damage and aberrant angiogenesis.

## Materials and methods

### Murine hindlimb ischemia model

The hindlimb ischemia model was performed as previously described [17,18]. All procedures were approved by the University of New South Wales Animal Care and Ethics Committee (Sydney, Australia; 07/26B), and conformed to the NIH Guide for the Care and Use of Laboratory Animals. Female mice (*n* ≥ 5, 8–12 weeks of age, 20–22 g) were anesthetized using isoflurane (2–3%). After shaving and cleaning the surgical area, a ligation was performed at the proximal and distal ends of the right femoral artery, and all the branches were dissected freely. To provide a parallel control, a sham procedure was conducted on the other hindlimb. Three days after hindlimb ischemia, the mice were euthanized by cardiac exsanguination. In this model, angiogenesis occurs predominantly in the ischemic distal bed [17,18], hence the gastrocnemius muscles were collected and split into two parts: (i) snap frozen for gene expression and (ii) fixed for histological analysis.

### Immunofluorescence and immunohistochemistry staining of murine tissue

Paraffin-embedded sections (5 μm) of gastrocnemius muscle from five ischemic and five non-ischemic tissues were probed for FKBPL (1:100, Proteintech, USA, cat. #100601AP), HIF-1α (1:200, Abcam, UK, cat. #179483) and VE-cadherin (1:200, Abcam, UK, cat. #33168). To measure capillary density, CD31 immunostaining (1:50, Abcam, UK, cat. #ab9498) was performed. Haematoxylin and eosin (H&E) staining was used to assess tissue architecture. At least five images (20X magnification) from each section were captured using an Olympus BX51 microscope.

### Cell culture

Human microvascular endothelial cells (HMEC-1) obtained from the Centre for Disease Control and Prevention (MTA M1224I) were maintained in MCDB 131 medium (Gibco, USA) supplemented with 10% foetal bovine serum (FBS, Bovogen biologicals, SFBS-AU), epidermal growth factor (0.01 µg/mL, Life technologies, cat. #PHG6045), penicillin/streptomycin (5 U/mL; Sigma Aldrich, P433), L-glutamine (2 mmol/L; Thermo Fisher, cat. #325030081) and hydrocortisone (500 µg/mL; Sigma Aldrich, cat. #H0888). The cells were incubated in a 37 °C humidified atmosphere with 5% CO_2_. Cells were not used beyond passage 26.

### Macrophage-conditioned medium

Human monocytes were isolated from buffy coats from healthy donors (Australian Red Cross Blood Service) as previously described [19]. All procedures were conducted in accordance with the Sydney Local Health District Human Ethics Committee (Reference X9.5/JUL17; Sydney, Australia). Monocytes were seeded in 6-well plates at a density of 1 × 10^6^ cells/well and differentiated to human monocyte–derived macrophages (HMDMs) over 10 days in RPMI 1640 medium (MilliporeSigma) supplemented with 10% (v/v) FBS. HMDMs were exposed to 10 ng/mL of IFN-ɣ (MilliporeSigma) and the macrophage-conditioned medium (MCM) was collected 48 h later and stored at -80 °C until use.

### HMEC-1 siRNA transfection and treatments

HMEC-1 cells were seeded onto 12-well plates at a density of 100,000 cells/well before being serum-arrested in MCDB 131 medium containing 0.1% (v/v) FBS for 24 h. The following day, cells were transfected with FKBPL targeted siRNA (24 µM, Dharmacon, USA) ± AD-01 (100 nM, NH2-QIRQQPRDPPTETLELEVSPDPAS-OH, Sigma-Aldrich, cat. #SML0928) or non-targeted siRNA, using Lipofectamine^®^ RNAiMAx reagent protocol (Invitrogen, USA, cat. #13778030). To mimic hypoxic or inflammatory conditions, cells were treated with dimethyloxalylglycine (DMOG, 1 mM, Sigma-Aldrich, USA, cat. #D3695) ± AD-01 (100 nM) or 50% (v/v) MCM ± AD-01 for various durations (24 h, 48 h and 72 h).

### Western blotting

Total protein was extracted using RIPA lysis buffer (50 mM Tris-HCL, 150 mM NaCl, 0.1% Triton, 0.5% Sodium deoxycholate, 0.1% SDS, pH = 8) containing 1% Halt Protease Inhibitor Cocktail (Thermo Fisher Scientific, USA). Proteins were separated using Mini**-**PROTEAN TGX Stain Free Gels (BioRad, cat. #4568084) and transferred onto nitrocellulose membrane (Bio-Rad, USA). Membranes were blocked with 5% skim milk for 3 h at room temperature and then probed with mouse anti-FKBPL (1:1000, Proteintech, USA, cat. #663891Ig) and rabbit anti-Glyceraldehyde 3-phosphate dehydrogenase (GAPDH, 1:8000, Abcam, UK, cat. #ab37168) antibodies. Membranes were incubated with their corresponding secondary antibody, anti-mouse IgG (1:10000, GE Healthcare, UK, cat. #NXA931) or anti-rabbit (1:8000, Abcam, UK, cat. #ab6721) antibody and imaged by chemiluminescence using a ChemiDoc MP imaging system (Bio-Rad, USA). Protein expression was quantified using ImageJ.

### Proteomics

Briefly, 10 µg of HMEC-1 protein lysates was digested with 100 ng of sequencing grade trypsin (Promega, USA) and enriched using STop-And-Go-Extraction tips (STAGE Tips). For elution of peptides, 100 µL of 5% ammonium hydroxide/80% acetonitrile was added to each tip and samples were collected in autosampler vials (Thermo Fisher, USA) and dried using SpeedVac vacuum concentrator (Thermo Fisher) at 40 °C for one hour. Finally, dried peptide samples were resuspended in 20 µL of 0.1% formic acid (final concentration 0.5 µg/µL) and analysed via LC-MS/MS as previously described [20] and provided in supplementary information.

The MS/MS data files generated using extracted samples were searched in PEAKS Studio X + (Bioinformatics solution) against the human proteome database (2019). The results of the search were then filtered to include peptides with a − log_10_P score that was determined by the false discovery rate (FDR) of < 1%, in which decoy database search matches were < 1% of the total matches. Principal component analysis (PCA) was performed using the top 500 variable proteins. Differential enrichment analysis was performed using the limma package (v3.54.2) [21] for DMOG vs. DMOG + AD-01 vs. control comparison. Significant proteins were defined at an FDR < 0.05. Enriched pathways in AD-01 or stimulus treatment groups were identified by ranking the proteins into descending log2 ratio in individual comparison and performing gene set enrichment analysis (GSEA) using clusterProfiler (v4.6.2) [22,23] through all pathways in KEGG database [24]. Significantly enriched pathways were defined as p-value < 0.05. Additional experimental details including materials, reagent, LC-MS/MS settings and data analysis are provided in the supplementary information.

### Real-time quantitative polymerase chain reaction (RT-qPCR)

RNA was extracted from snap frozen gastrocnemius muscle tissues as previously described [18]. RT-qPCR was performed using CFX96 thermocycler (Bio-Rad, USA) and SensiFast™ SYBR^®^ No-Rox one-step kit (Bioline, Australia, cat. #72001) in triplicates. Relative changes in gene expression between groups were normalised using the 2^− ΔΔCt^ method to β-actin. The sequences for mouse primers are as follows: β-Actin (forward 5′-GATGTATGAAGGCTTTGGTC-3′ and reverse 5′-TGTGCACTTTTATTGGTCTC-3′), FKBPL (forward 5′-TCTCTCAGGGATCAGGAG-3′ and reverse 5′-TATTTAAGATTTGCTGGGCG-3′), ICAM (forward 5′-CAGTCTACAACTTTTCAGCTC-3′ and reverse 5′-CACACTTCACAGTTACTTGG-3′), VCAM (forward 5′-ACTGATTATCCAAGTCTCTCC-3′ and reverse 5′-CCATCCACAGACTTTAATACC − 3′).

### 3D microfluidic endothelial cell model

The microfluidic tissue culture devices were obtained from AIM Biotech (Singapore). These devices are made up of cyclic olefin polymer chip bodies laminated in highly gas permeable laminates. Each device consisted of three microfluidic sites, each with a central gel region with a width of 1.3 mm and a height of 0.25 mm, flanked by two parallel channels for the media (0.5 mm width). Interstitial flow in the device is generated by adding different volumes to opposing channels, resulting in a pressure gradient. ECM solution was prepared using collagen type I (2.5 mg/mL, Gibco™, cat. #A1048301), 10X PBS, H_2_O, and NaOH (0.5 N) at pH = 7.4 and kept on ice to prevent polymerization. The ECM solution was injected into the gel channel according to the manufacturer’s protocols [25] and incubated at 37 °C and 5% CO_2_ for 40 min to allow gel polymerization via thermal cross-linking. Next, the media channels were primed with 10 µL of MCDB 131 medium for 5 min, after which the volume was increased to 120 µL. HMEC-1 cells were seeded onto the left media channel at 20,000 cells/channel and serum-arrested for 24 h in MCDB 131 medium supplemented with 0.1% (v/v) FBS prior to exposure to different treatment condition: (i) 24 µM FKBPL targeted siRNA, (ii) 1 mM DMOG, (iii) 50% (v/v) MCM, ± 100 nM AD-01. A non-targeted siRNA treatment and untreated cells were used as controls. To establish a chemoattractant gradient for cell migration, 50% (v/v) FBS was added to the right media channel. The microfluidic devices were then incubated at 37 °C and 5% CO_2_ for 72 h and media were refreshed every 24 h.

### Immunofluorescence staining

The microfluidic devices were washed with PBS after the cell culture medium was removed from the media channels. HMEC-1 cells in collagen were fixed with 4% paraformaldehyde (PFA; Sigma-Aldrich, USA) for 15 min at 37 °C and permeabilized with 0.1% Triton‐X (Sigma‐Aldrich, USA), for 10 min at room temperature. The cells were probed with FKBPL (1:200, Proteintech, USA, cat. #100601AP), CD31 (1:200, Abcam, UK, cat. #ab24590), HIF-1α (1:100, Abcam, UK, cat. #179483) and VE-cadherin (1:100, Abcam, UK, cat. #33168) and incubated at 4 °C overnight. Following washing, goat anti-rabbit IgG H&L (Alexa Fluor^®^ 488, Abcam, cat. #150077) and goat anti-mouse IgG H&L (Alexa Fluor^®^ 594, Abcam, cat. #150116) secondary antibodies were added for 2 h at room temperature. The cell nuclei were stained with DAPI (10 µg/mL, Invitrogen, USA, cat. #D1306) for 1 h at room temperature. Washed microfluidic devices were stored at 4 °C.

### Widefield and laser scanning confocal microscopy

Fluorescence images were acquired using Nikon TIE2 widefield fluorescence and transmitted light microscope with a 20X objective (NA 0.75) and long working distance (2300 μm) with optical slices of 1 μm. Microfluidic device images were either deconvolved with NIS-Elements (version 5.3) using the Richardson-Lucy method or clarified using NIS-Elements Clarify.ai [26]. High-resolution images were obtained using a Leica Stellaris confocal microscope with a 20X objective (NA 1.45) with Nyquist sampling and Z stacks (0.2 μm optical slices) using a 1 AU pinhole. At least five images were taken from each site of the microfluidic device.

### Quantification of immunofluorescence

At least five scanned images from tissue samples or microfluidic devices were imported as tiff files to the quantitative microscopy-based image analysis system StrataQuest (version 7.1.1.121, TissueGnostics GmbH, Vienna, Austria), which assesses protein expression at the single cell level [27–29]. The staining intensities were determined as gray values between 0 and 255 for 8-bit images and 0 and 65,500 for 16-bit images and were normalised to the number of cells and the image area. The nuclei were identified within the DAPI channel using the deep neural network (DNN) module within StrataQuest. A secondary measurement mask was created as an extension to the identified nuclei to perform single cell analysis. The mean staining intensity in the cytoplasm or in the nucleus was assessed after setting a cut-off associated with the internal negative control. In the case of the migration analysis, specific gates were created within the region of interest to quantify the number and protein expression, of cells that migrated from the microfluidic channel.

### Statistical analysis

The results obtained with murine samples, and in vitro 2D and 3D microfluidic experiments are presented as the mean ± SEM. To test for normality of the data, a Shapiro-Wilk test was performed followed by a two-tailed unpaired t-test or one-way ANOVA with post-hoc multiple comparison tests. The Mann-Whitney or Kruskal-Wallis test was used for not normally distributed data. Statistical analysis was conducted using GraphPad Prism (version 8.4.3 software, USA) and a p-value < 0.05 was considered statistically significant.

## Results

### Ischemia suppresses FKBPL expression and stimulates angiogenesis in gastrocnemius muscle

**Fig. 1:**
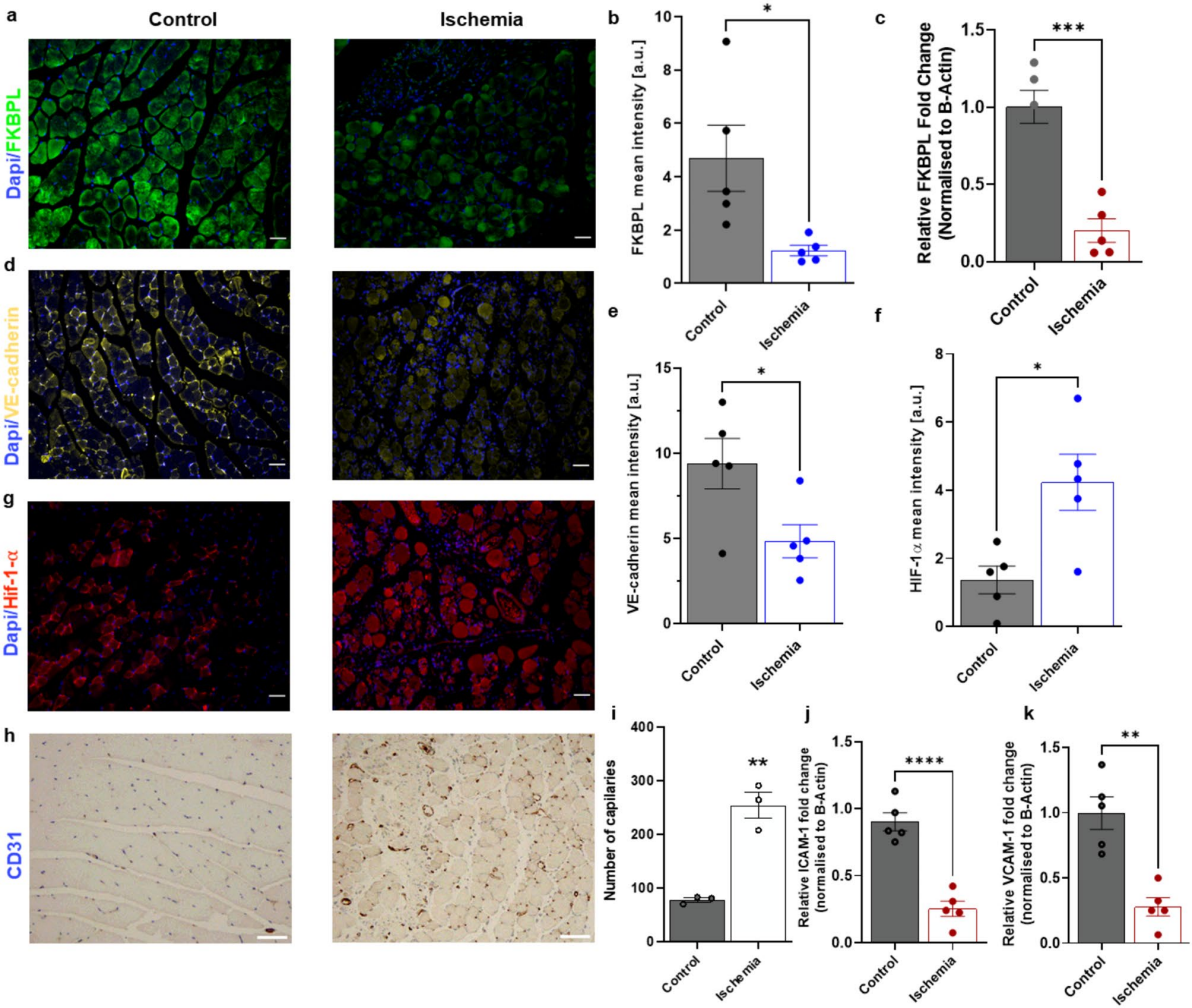
FKBPL expression is suppressed in ischemia. Gastrocnemius muscle tissue from ischemic leg and non-ischemic leg (control) was processed for immunofluorescence/immunohistochemistry staining or RNA extraction. **(a)** Representative immunofluorescent images of FKBPL; **(b)** Protein (green) and **(c)** mRNA, expression of FKBPL was quantified based on mean fluorescence or fold-change normalised to β-Actin, respectively. **(d)** Representative immunofluorescence images of VE-cadherin (yellow); protein expression of VE-cadherin **(e)** and HIF-1 α **(f)** were quantified based on mean fluorescence; **(g)** representative immunofluorescence images of HIF-1 α. DAPI represents cell nuclei. **(h)** Representative immunohistochemistry staining of CD31 (blue). **(i)** Capillary density was quantified and compared between the two groups. (**j**, **k**) qPCR analysis showed a significant downregulation of ICAM-1 and VCAM-1 with ischemia. mRNA expression was normalised to β-Actin. Scalebars represent 100 μm. Data are mean ± SEM, *n* = 3–5, unpaired student’s t-test, *p < 0.05, **p < 0.01, ***p < 0.001, ****p < 0.0001

In the hindlimb ischemia model on day 3, FKBPL protein expression in the ischemic gastrocnemius muscle was significantly reduced (~ 3.8 fold, *p* = 0.02), at the single cell level (*p* = 0.008, Fig. 1a, b); this change was associated with reduced mRNA expression (*p* < 0.001; Fig. 1c). VE-cadherin expression was also significantly lower in the ischemic limb (*p* = 0.033, Fig. 1d, e), whereas HIF-1α expression was increased ~ 4-fold (*p* = 0.014, Fig. 1f, g), compared to that in the non-ischemic control. Revascularisation was then evaluated in ischemic tissue, and CD31 staining revealed ~ 30% increase in the number of new capillaries compared to control (*p* = 0.002, Fig. 1h, i). To assess markers of leukocyte infiltration and inflammation during hindlimb ischemia [30,31], ICAM-1 and VCAM-1 mRNA expression was determined. The expression of both genes was reduced by ~ 3.6-fold in the ischemic gastrocnemius muscle compared to control (ICAM: *p* < 0.0001, Fig. 1j; VCAM-1: *p* = 0.001, Fig. 1k). Collectively, these results suggest that ischemia stimulates angiogenesis through inhibition of FKBPL, which is associated with a reduction in the endothelial cell integrity marker, VE-cadherin, and the adhesion markers, VCAM-1 and ICAM-1.

### AD-01 abrogates hypoxia- and inflammation-induced HIF-1α and FKBPL changes in protein expression in 3D microfluidic cell culture model

**Fig. 2:**
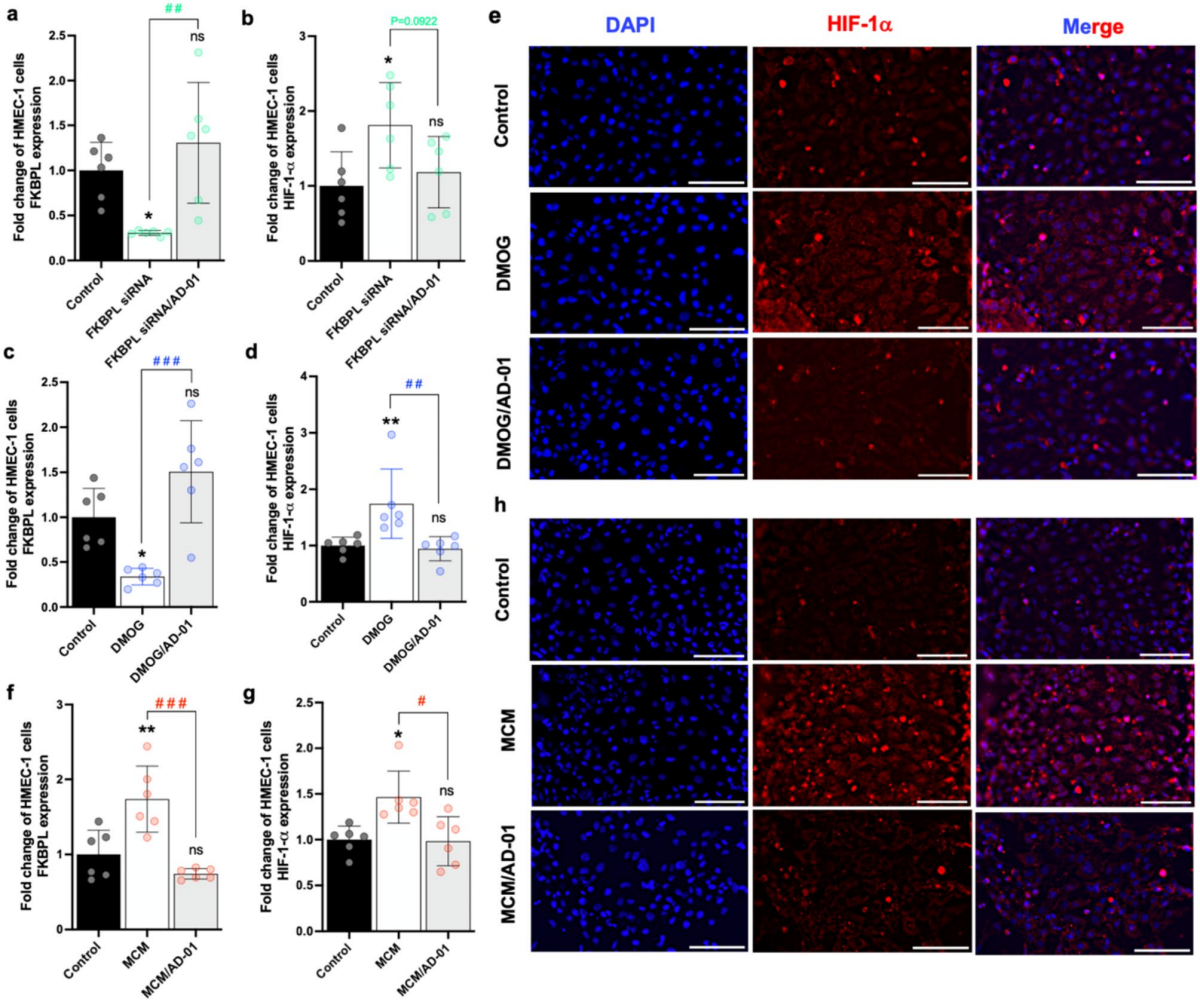
AD-01 attenuates changes in HIF-1α and FKBPL protein expression in HMEC-1 cells in a 3D microfluidics model. In the 3D microfluidic settings, collagen matrix (2.5 mg/mL) was added to the central channel and HMEC-1 cells were treated with 24 µM FKBPL siRNA, 1 mM DMOG or 50% MCM, ± 100 nM AD-01, for 72 h. Chips were probed with antibodies and subjected to immunofluorescence imaging to determine the expression of FKBPL, HIF-1α and DAPI. Knockdown of FKBPL using siRNA significantly decreased **(a)** FKBPL expression, and **(b)** increased HIF-1α protein expression in HMEC-1 cells, while AD-01 normalised FKBPL protein expression only in hypoxic conditions. As control for siRNA treatment, non-targeted siRNA was used. DMOG significantly reduced and increased **(c)** FKBPL and **(d)** HIF-1α protein expression, respectively, and AD-01 restored the expression of both proteins to levels similar to control. **(e)** Representative images of HIF-1α immunofluorescence staining in the presence of DMOG ± AD-01. The expression of **(f)** FKBPL and **(g)** HIF-1α protein in HMEC-1 cells is increased by MCM, however after 72 h treatment with AD-01, the expression of both proteins was normalised. **(h)** Representative images of HIF-1α immunofluorescence staining in the presence of 50% MCM media ± AD-01. Scale bars represent 100 μm. The data are plotted as the mean ± SEM; every data point represents one microfluidic device containing three experimental units; *n* = 3. The data passed the Shapiro-Wilk normality test and were analyzed by one-way analysis of variance (ANOVA) with Tukey’s post-hoc test, *p < 0.05, **p < 0.01, ***p < 0.001 (compared to control); ^#^p<0.05, ^##^p<0.01, ^###^p<0.001 (compared to stimuli conditions)

Using 2D HMEC-1 cell culture we optimised the time course to determine the impact of exposure to hypoxic/inflammatory stimuli on FKBPL mechanisms, with or without AD-01 treatment. This was further validated through siRNA-based experiments. No significant changes with AD-01 treatment were observed at the 24 h and 48 h time point for any of the conditions (Supplementary Fig. 1). Silencing FKBPL with siRNA inhibited protein expression by ~ 60% and ~ 70% after 48 and 72 h, respectively (48 h: *p* = 0.0008; 72 h: *p* = 0.015; Supplementary Fig. 1a), but not at 24 h (Supplementary Fig. 1a). Importantly, FKBPL suppression was restored to control levels with AD-01 at 72 h (*p* = 0.010; Supplementary Fig. 1a). Similar to the FKBPL siRNA treatment, FKBPL protein expression was suppressed by ~ 70% (*p* = 0.022; Supplementary Fig. 3b) after 72 h of DMOG exposure. Remarkably, AD-01 recovered FKBPL expression after 72 h of hypoxia (*p* = 0.003; Supplementary Fig. 1b). Although MCM had no effect on FKBPL expression at 24 h (Supplementary Fig. 1c), MCM significantly increased the expression of FKBPL at 48 and 72 h, whereas AD-01 had no additional impact on FKBPL protein expression (48 h; *p* = 0.009; 72 h: *p* = 0.0013, Supplementary Fig. 1c). Taken together, these data show that hypoxia represses FKBPL, while inflammation increases FKBPL protein expression at 72 h, and that AD-01 rescues hypoxia-induced FKBPL suppression without impacting on FKBPL expression under inflammatory conditions. HIF-1α plays an important role in angiogenesis by stimulating the expression of VEGF, and promoting the restoration of oxygen in ischemic tissues, however, it is also associated with inflammation in atherosclerotic lesions [32,33]. The role of FKBPL in hypoxia/HIF-1α- or inflammation-mediated effects on endothelial cells has not been previously established. Using a 3D microfluidics model, we found that suppressing FKBPL expression with a siRNA pool (Fig. 2a and Supplementary Fig. 2) increased HIF-1α protein expression (*p* = 0.04; Fig. 2b and Supplementary Fig. 3). Although, AD-01 restored the expression level of FKBPL to those observed in the control group (*p* = 0.003, Fig. 2a), it had no significant effect on HIF-1α protein expression (Fig. 2b; *p* = 0.09) caused by endogenous FKBPL knockdown. DMOG treatment significantly decreased FKBPL expression in HMEC-1 cells by ~ 3-fold compared to control, while AD-01 rescued the FKBPL protein expression after 72 h (control *p* = 0.0003, Fig. 2c and Supplementary Fig. 4a). As expected, HIF-1α protein expression was significantly greater in HMEC-1 cells following 72 h treatment with DMOG compared to the control (Fig. 2d, e). Remarkably, AD-01 restored HIF-1α protein expression to normal (*p* = 0.004; Fig. 2d, e). Inflammation led to a significant increase in FKBPL protein expression in HMEC-1 cells compared to the control (Fig. 2f and Supplementary Fig. 4b). In contrast to the results of the 2D in vitro studies, the expression of the FKBPL protein was abrogated by AD-01 in the 3D microfluidics model (*p* = 0.0002; Fig. 2f and Supplementary Fig. 4b). These findings suggest a potential negative feedback mechanism by which AD-01 regulates endogenous FKBPL protein expression under inflammatory conditions. Treatment of HMEC-1 cells with MCM for 72 h resulted in higher HIF-1α expression than control, and AD-01 was able to normalise HIF-1α expression (control *p* = 0.005; Fig. 2g, h). Taken together, these data indicate that FKBPL is an important new mechanism regulating HIF-1α signalling under hypoxic and inflammatory conditions.

### AD-01 attenuates hypoxia-induced HMEC-1 cell migration and improves vascular integrity

**Fig. 3:**
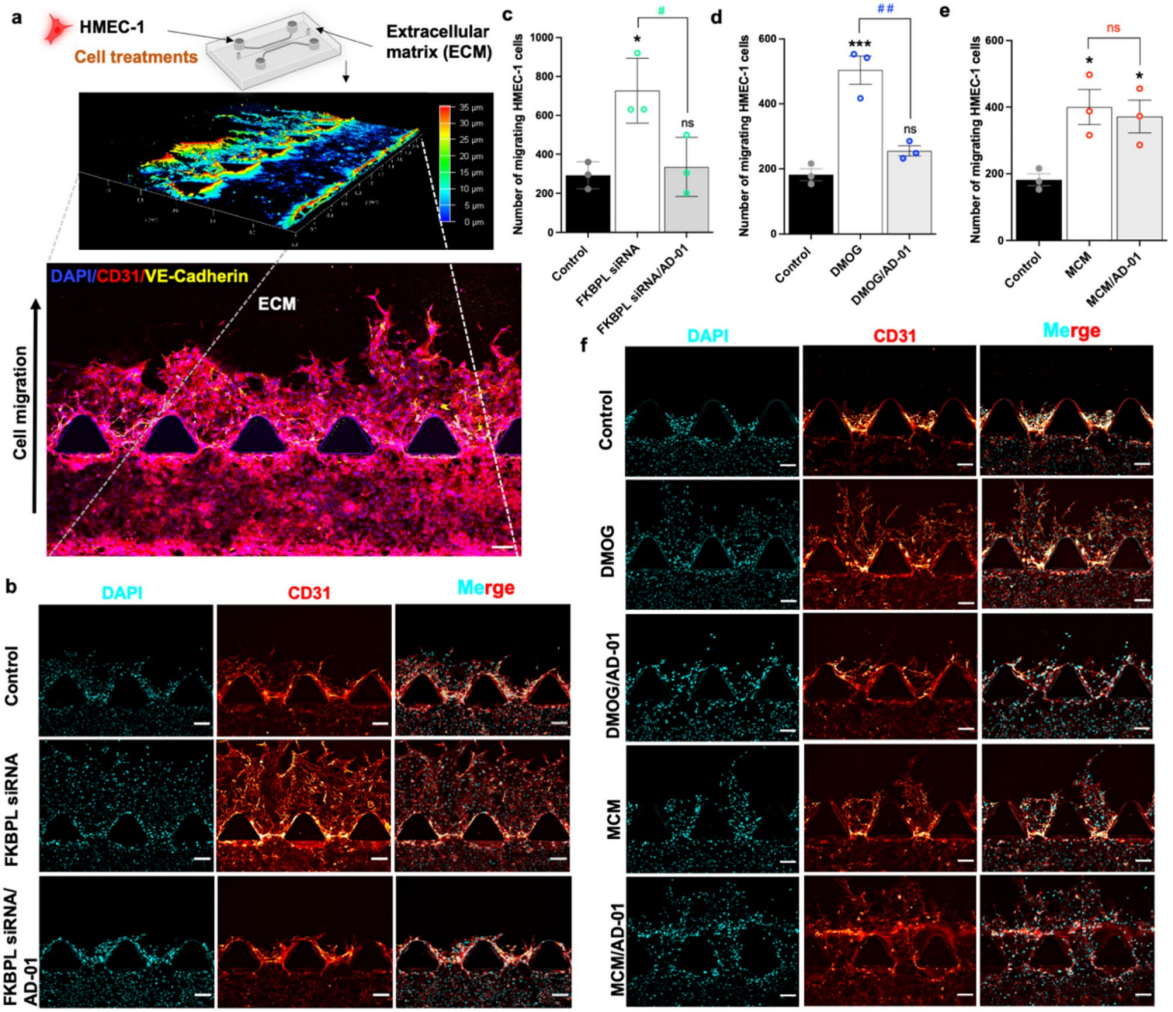
Hypoxia and inflammation presence modify HMEC-1 migration. (**a**) Top panel, representative image depicting the 3D microfluidics device design. A collagen matrix (2.5 mg/mL) was added to the central channel and HMEC-1 cells were added to the side channel. Middle panel, 3D-rendering of the microfluidic device. Bottom panel, higher-power magnification image showing cell migration. A gradient of chemoattractant for cell migration was established by adding 50% (v/v) fetal bovine serum (FBS) to the right media channel. Chips were treated with 24 µM FKBPL siRNA or 1 mM DMOG or 50% MCM, ± 100 nM AD-01. After 72 h, chips were probed with antibodies and subjected to immunofluorescence imaging to determine the expression of CD31 and DAPI. As control for siRNA treatment, non-targeted siRNA was used. Untreated cells were used as controls for the DMOG and MCM treatments. (**b**) Representative images of CD31 and DAPI stained HMEC-1 following FKBPL siRNA treatment ± AD-01. (**c**) FKBPL siRNA, (**d**) DMOG and **(e)** MCM treatment significantly increased HMEC-1 migration whereas AD-01 suppressed only siRNA FKBPL- and DMOG-induced migration. **(f)** Representative images of CD31 and DAPI stained HMEC-1 cells following DMOG/MCM treatment ± AD-01. Scale bars represent 100 μm. The data were plotted as the mean ± SEM; every data point represents one microfluidic device containing three experimental units, *n* = 3. The data passed the Shapiro-Wilk normality test and were analyzed by one-way ANOVA with Tukey’s post-hoc test; *p < 0.05, ***p < 0.001 (compared to control); ^#^p<0.05, ^##^p<0.01 (compared to stimuli conditions)

Endothelial cell migration is an essential step in angiogenesis. Therefore, we investigated cell migration in vitro using 3D microfluidic devices as a functional consequence of endothelial cells under hypoxic/HIF-1α activation and inflammatory conditions using MCM, with or without AD-01 treatment (Fig. 3a).

**Fig. 4:**
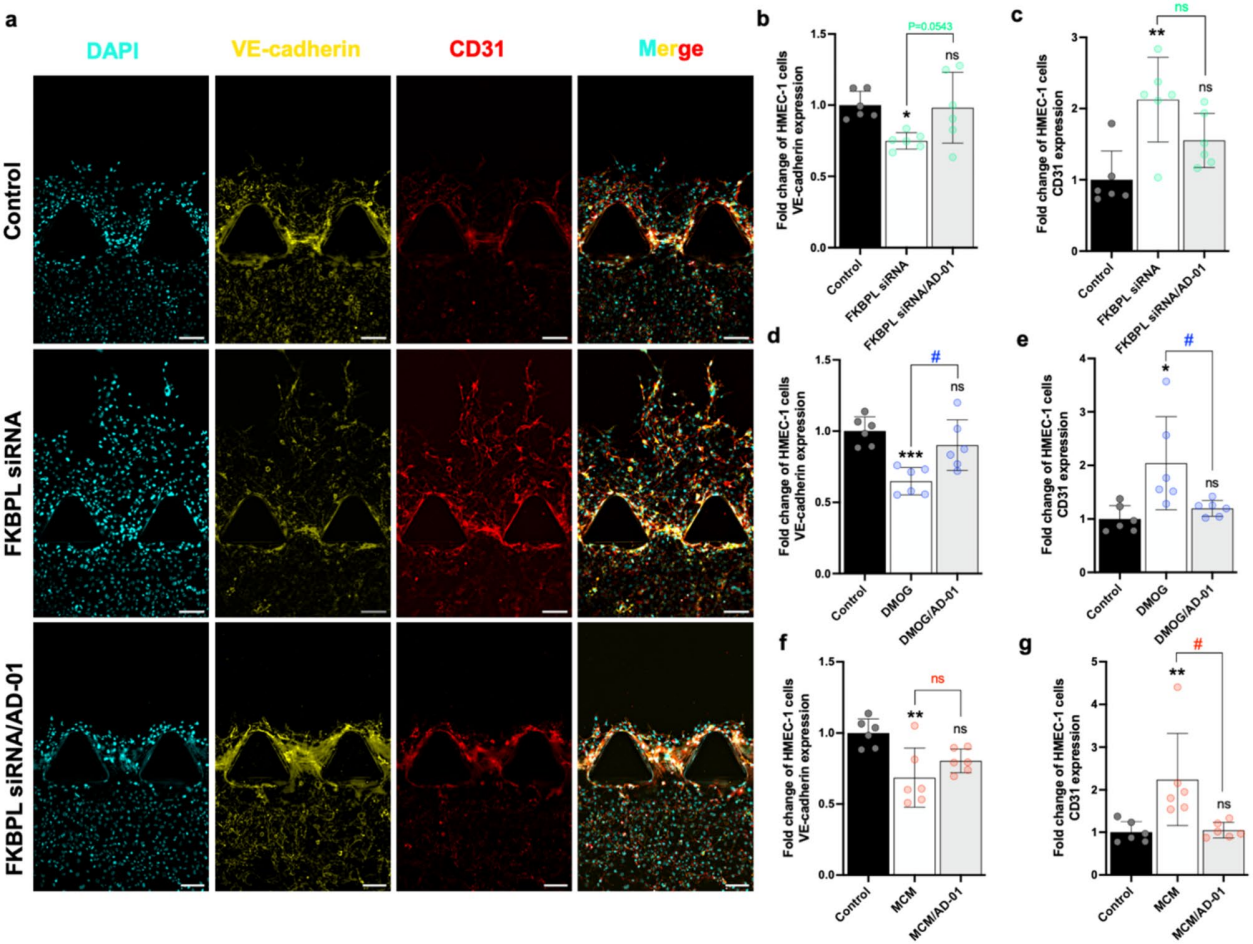
The protein expression of VE-cadherin and CD31 in HMEC-1 cells is modulated by FKBPL siRNA, DMOG, and MCM treatment. 3D microfluidic devices containing HMEC-1 cells were treated with 24 µM siRNA, 1 mM DMOG and 50% MCM, ± 100 nM AD-01, for 72 h. The chips were subsequently probed with antibodies and subjected to immunofluorescence imaging to determine the expression of VE-cadherin, CD31, and DAPI. SiRNA control - non-targeted siRNA. DMOG, MCM control - untreated cells. (**a**) Representative images of VE-cadherin and CD31 staining following FKBPL siRNA ± AD-01 treatment. FKBPL siRNA significantly reduced and enhanced the expression of the **(b)** VE-cadherin and **(c)** CD31 proteins in HMEC-1 cells, respectively, while AD-01 restored the expression of VE-cadherin, although the difference was not statistically significant (*p* > 0.05). DMOG decreased and increased the expression of the **(d)** VE-cadherin and **(e)** CD31 proteins, respectively, in HMEC-1 cells and AD-01 restored the expression of both proteins. MCM treatment resulted in significant **(f)** decrease in VE-cadherin and **(g)** increase in CD31, protein expressions, while AD-01 treatment restored only the expression of the CD31 protein. Scale bars represent 100 μm. The data were plotted as the mean ± SEM, every data point represents one microfluidic device containing three experimental units; *n* = 3, The data passed the Shapiro-Wilk normality test and were analyzed by one-way ANOVA with Tukey’s post-hoc test, *p < 0.05, **p < 0.01, ***p < 0.001 (compared to control); ^#^p<0.05 (compared to stimuli conditions)

FKBPL knockdown by siRNA resulted in a significant increase in HMEC-1 migration compared to that in the control; however, after 72 h with AD-01 treatment, HMEC-1 migration completely returned to control levels (*p* = 0.014; Fig. 3b, c). As expected, DMOG-stimulated HMEC-1 migration, which was also attenuated by AD-01 (*p* = 0.0005; Fig. 3d). MCM increased HMEC-1 migration, however, AD-01 had no effect (*p* = 0.022; Fig. 3e, f). In most vascular beds, VE-cadherin and CD31 play critical roles in controlling endothelial barrier function and properties, and, if aberrant, can lead to various vascular pathologies [34, 35]. FKBPL siRNA significantly decreased VE-cadherin protein expression, whereas CD31 expression was significantly increased. AD-01 treatment appeared to normalize the expression of these proteins within 72 h in HMEC-1 cells, however the difference between the FKBPL siRNA vs. FKBPL siRNA/AD-01 groups were not statistically significant (Fig. 4a-c). Similarly, hypoxia also resulted in a reduction in VE-cadherin protein expression and stimulated CD31 protein expression in HMEC-1 cells. The expression of these proteins were effectively restored within 72 h upon treatment with AD-01 (VE-cadherin: *p* = 0.0009, Fig. 4d, Supplementary Fig. 1; CD31: *p* = 0.009, Fig. 4e, Supplementary Fig. 5). MCM-induced inflammatory conditions had similar effects on CD31 and VE-cadherin expression; however, AD-01 normalized MCM-induced increase in CD31 but not VE-cadherin, protein expression in HMEC-1 cells after 72 h (VE-cadherin: *p* = 0.006; Fig. 4f and Supplementary Fig. 6; CD31: *p* = 0.0004; Fig. 4g and Supplementary Fig. 6).

### AD-01-mediated endothelial cell mechanisms target tissue remodelling and vascular integrity mechanisms in hypoxia

**Fig. 5:**
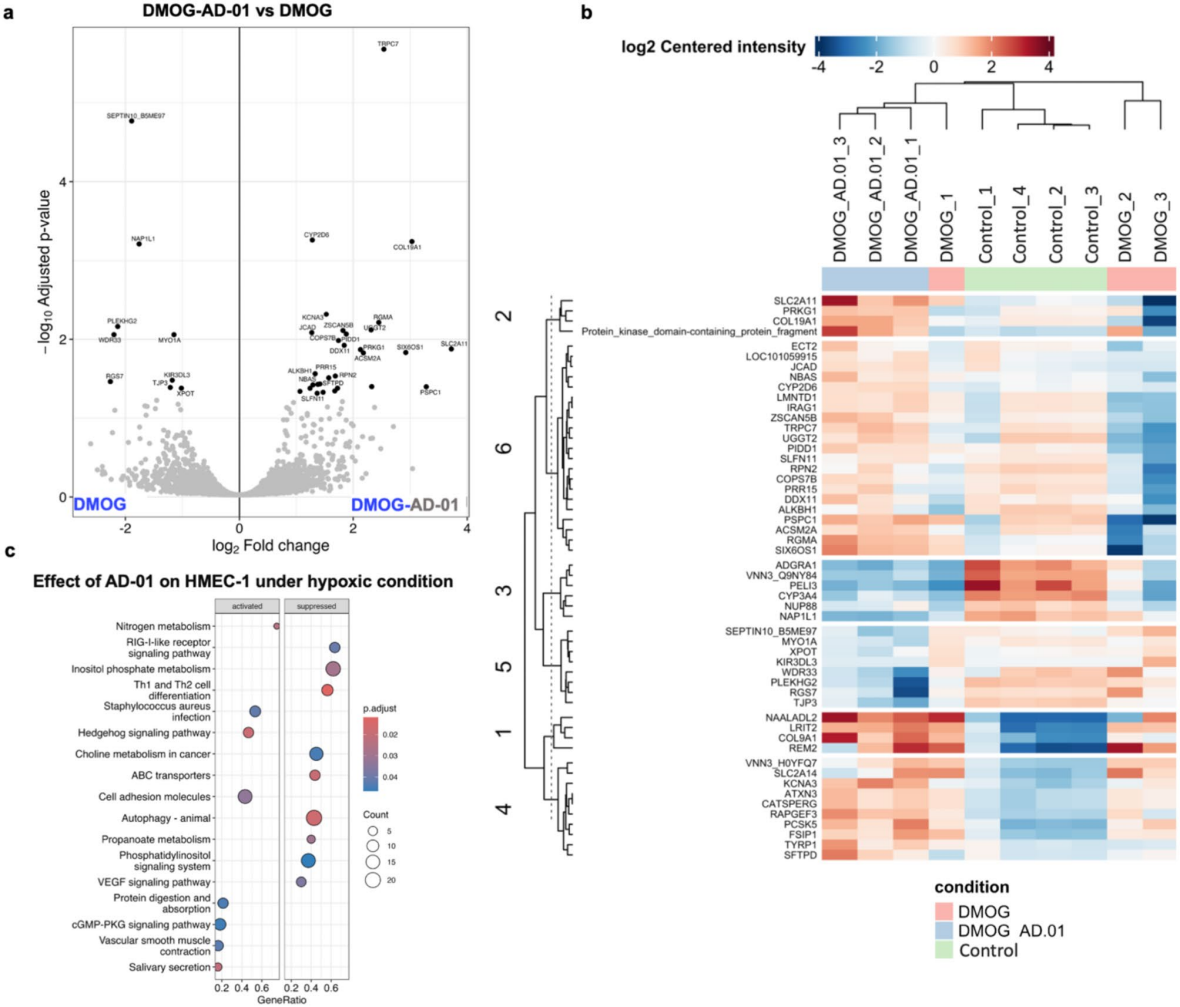
Proteomic profile and pathway analysis of the mechanisms involved in AD-01-mediated effect on HMEC-1 cells under hypoxic conditions. Protein lysates were extracted from HMEC-1 cells after 72 h of treatment with 1 mM DMOG, either with or without 100 nM AD-01 treatment and subjected to LC-MS/MS analysis. (**a**) Differential enrichment analysis comparing DMOG vs. DMOG + AD-01. (**b**) Heatmap of the total number of significant proteins in HMEC-1 cells treated with DMOG, DMOG + AD-01 or the control (untreated cells). (**c**) Comparison of the 17 pathways enriched in HMEC-1 cells treated with DMOG ± AD-01

Given that AD-01 has a therapeutic potential for restoring hypoxia-induced changes in endothelial cell function without impacting the influence of MCM, we wanted to further explore the mechanisms of action of AD-01 relevant to hypoxic conditions alone. Label-free proteomic analysis was performed on: (1) Control, (2) DMOG and (3) DMOG + AD-01, treated HMEC-1 protein lysate samples, and the relative abundance of tryptic peptides was measured. The majority of proteins (> 85%) were consistently detected across most of the samples (Supplementary Fig. 7a and b). Dimensionality reduction of the top 500 highly variable proteins revealed relatively consistent and biologically meaningful clustering of the samples according to their corresponding conditions, and PCA showed that all the samples had heterogeneous proteomes (Supplementary Fig. 7c and Fig. 8). In total, 10 differentially abundant proteins were detected between the DMOG-treated and control groups (Supplementary Fig. 9a and supplementary Table 1); 38 differentially abundant proteins were identified in the DMOG vs. DMOG + AD-01 comparison (Fig. 5a and Supplementary Table 2).

In DMOG-treated HMEC-1 cells, AD-01 significantly altered the expression of proteins involved in cellular processes regulating cell division, vesicles trafficking, and protein translocation (Fig. 5a, b). DMOG + AD-01 treatment significantly reduced the expression of unconventional myosin-1α (fold change=-1.15; *p* = 0.009), while enhancing the expression of the NBAS subunit of NRZ tethering complex (Fold change = 1.42; *p* = 0.04) and the expression of dolichyl-diphosphooligosaccharide-protein glycosyltransferase subunit 2 (Fold change = 1.69, *p* = 0.03 (Supplementary Table 2). Moreover, AD-01 significantly increased the expression of proteins involved in tissue remodelling and vascular integrity including the collagen alpha-1(XIX) chain protein (fold change = 3.03; *p* = 0.0005) and junctional cadherin associated-5 protein (JCAD) (fold change = 1.27; *p* = 0.008) (Supplementary Table 2). A total of 17 pathways were significantly altered between the DMOG and DMOG + AD-01 groups (Fig. 5c). AD-01 reduced the expression of pro-inflammatory transcription factor NF-κB and inhibited the RIG-I-like receptor and VEGF-signalling pathways, respectively (Fig. 5c). These findings are significant because they are involved in hypoxia-induced angiogenesis [36–38]. AD-01 also activated the cGMP-PKG and cell adhesion molecule pathways, which may, in part, help prevent the onset of acute hypoxia [39,40].

These data suggest that AD-01 may constitute a novel therapeutic approach for the treatment of hypoxia-induced diseases in which endothelial dysfunction plays a role.

## Discussion

**Fig. 6:**
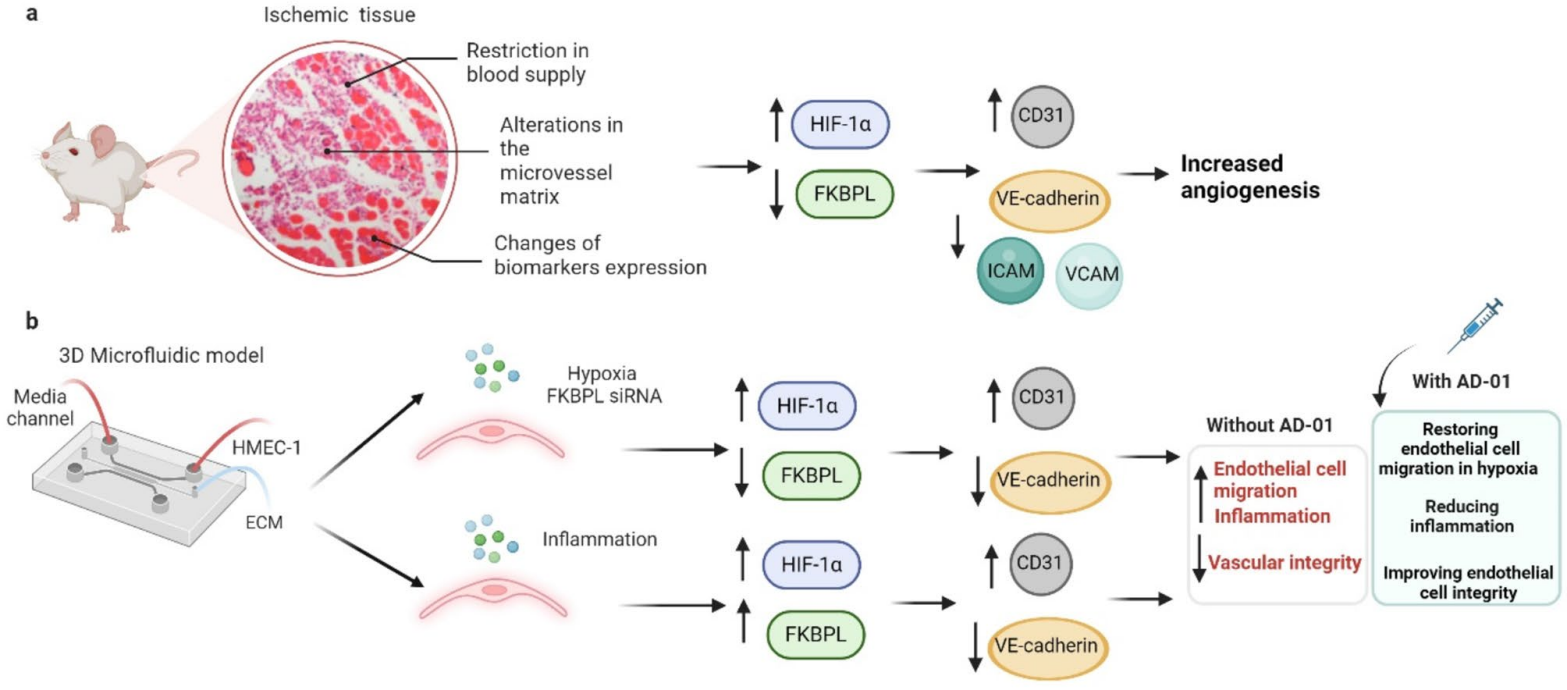
Summary of the endothelial cell specific role of FKBPL in association with HIF-1α in hypoxia and inflammation. (**a**) An in vivo wild-type mouse model of hindlimb ischemia demonstrated a notable reduction in FKBPL expression, accompanied by altered expression of HIF-1α, VE-cadherin, and CD31, resulting in compromised vascular integrity. (**b**) AD-01 demonstrated therapeutic potential by targeting HIF-1α, which restored endothelial cell function, maintained vascular integrity, and alleviated hypoxia and inflammation-induced endothelial cell damage. Created in https://BioRender.com

In this study, we highlight the crucial role of FKBPL in regulating endothelial cell function and integrity under hypoxic and inflammatory conditions, using an in vivo mouse model of hindlimb ischemia, and an advanced in vitro 3D microfluidic model of human microvascular endothelial cell dysfunction (summarised in Fig. 6). Specifically, we showed the following three key findings: (i) FKBPL-based preclinical candidate peptide therapeutic, AD-01, normalised endothelial cell migration under hypoxic but not inflammatory conditions by decreasing HIF-1α and CD31, and increasing VE-Cadherin, protein expression, (ii) first demonstration linking FKBPL mechanism to ischemia in a mouse model of peripheral artery disease, and (iii) new knowledge on the mechanisms of action of AD-01 that apart from FKBPL, targets other anti-inflammatory, anti-angiogenic and vascular remodelling proteins through inhibiting NF-kB and increasing collagen alpha-1(XIX) chain and JCAD, protein expression, under hypoxic settings.

In our in vivo mouse model of hindlimb ischemia, surgical ligation of the femoral artery above the epigastrica and profunda femoris was performed to predominantly promote hypoxia-induced angiogenesis in gastrocnemius muscles [17,18]. However, studies have shown the presence of inflammation in similar hindlimb ischemia models, which is primarily due to ligation below the epigastrica and profunda without removing the femoral artery or ligation proximal to the popliteal artery [41,42]. As hypoxia is the primary driving force behind angiogenesis in this model likely through the regulation of chemokines, including CXCL12 and its receptor [17,19,43], we observed a significant increase in HIF-1α protein expression, which was positively associated with CD31 expressing capillaries. Importantly, ischemia-induced angiogenesis resulted in substantial downregulation of FKBPL protein/gene expression, in association with reduced VE-cadherin protein, ICAM, and VCAM gene, expression. Although the suppression of FKBPL appears to be an important mechanism leading to enhanced angiogenesis in ischemia/hypoxia, previous work in *fkbpl*^*+/−*^ mice showed that heterozygous knockdown of FKBPL, under physiological conditions, stimulated angiogenesis but with impaired vascular integrity and less robust blood vessels [12]. Similarly, reduced VE-cadherin expression has been shown to disrupt endothelial cell junctions and reduce vascular integrity [44]. In addition, ICAM and VCAM are predominantly stimulated during inflammation to facilitate the recruitment and adhesion of leukocytes to endothelial cells to form and stabilize blood vessels [45,46]. It remains to be determined whether the reduction in the expression of these vascular and inflammatory factors observed in our study is due to surgery, hypoxia, duration of ischemia or reduction in FKBPL expression. Overall, these data from our mouse model of hindlimb ischemia suggest that hypoxia, in conjunction with the upregulation of HIF-1α, may inhibit the expression of FKBPL, resulting in a pro-angiogenic response and a reduction in the integrity and stability of the new vasculature in ischemic tissues.

Similar to in vivo settings, in our 3D microfluidic models of microvascular endothelial cell dysfunction, FKBPL knockdown or hypoxia induction via DMOG treatment led to a significant increase in HIF-1α and CD31, while reducing VE-cadherin. The FKBPL-based therapeutic peptide mimetic, AD-01, was able to restore the expression of these proteins under hypoxic conditions. HIF-1α plays a critical role in regulating the adaptive response to hypoxia, however, aberrant HIF-1α expression can result in chronic inflammation, oxidative stress, impaired angiogenesis, and energy metabolism, hence contributing to the development of CVD [47–51]. Consequently, angiogenesis-targeting therapeutics including AD-01, which can reverse HIF-1α overexpression and preserve physiological angiogenesis in hypoxia-induced vascular damage are highly desirable.

We also showed that treatment with AD-01 under hypoxic conditions, can normalise CD31 expression and abrogate VE-cadherin downregulation in microvascular endothelial cells. This may result in improved angiogenic responses to ischemia and better perfusion recovery in patients with CVD, particularly in the context of peripheral artery disease. CD31 is a transmembrane glycoprotein that is enriched at endothelial cell intercellular junctions and mediates cell-cell adhesion between endothelial cells and adherent leukocytes. It also regulates endothelial cell functions including cell migration, tube formation, and angiogenesis [52–54]. Thus, dysregulation of CD31 expression in endothelial cells may result in impaired endothelium and disruption of endothelial junctions, which may exacerbate endothelial dysfunction. In vitro and animal studies have shown that reducing VE-cadherin at the junctions between endothelial cells decreases the integrity of endothelial cells through an increase in endothelial cell permeability, which facilitates the infiltration of inflammatory cells from blood vessels into tissues [55–57].

In our 3D microfluidic endothelial cell model of inflammation, we used 50% MCM to stimulate the expression of several chemokines and their receptors including CCL2, CCL5, and CX3CL1 as demonstrated previously [58], which have been shown to regulate inflammation-driven angiogenesis in the vascular system during the development and progression of CVD [58–61]. FKBPL has also been shown to regulate inflammation through STAT3, CD44, and NF-κB [16,62,63]. Although, previous work demonstrated that AD-01 requires CD44 to inhibit angiogenesis, it still modulated NF-κB expression in bone marrow derived macrophages in a CD44^−/−^ mouse model, indicating that AD-01 may regulate inflammation through a different pathway than CD44 [16]. While inflammation and hypoxia signalling pathways coexist within the vasculature, the impact of inflammation on HIF-1α remains unclear. Considering that AD-01 regulates HIF-1a expression, we expect that AD-01 might also have anti-inflammatory effects, which merits further investigation.

In contrast to hypoxia, inflammation resulted in an increase in FKBPL expression following 72 h of treatment with MCM. AD-01 abrogated the overexpression of FKBPL induced by MCM, but it was unable to reverse MCM-stimulated endothelial cell migration, in 3D microfluidic chips. A previous study demonstrated that AD-01 negatively regulates FKBPL in cardiomyoblasts after exposure to angiotensin-II for 48 h [64]. These findings suggested that AD-01 has a complex and compensatory mechanism for controlling endothelial cell FKBPL expression under hypoxic and inflammatory environments; mechanisms identified through proteomics include the inhibition of NF-kB and increased collagen alpha-1(XIX) chain and JCAD protein expression. Like hypoxia, MCM treatment also increased HIF- 1α and CD31, while reducing VE-cadherin. However, under inflammatory conditions, AD-01 was only capable of restoring the expression of HIF-1α and CD31 proteins, and did not restore the decreased VE-cadherin expression. These findings suggest that AD-01 may mitigate stress-induced changes in FKBPL through HIF-1α, although this effect appears to be primarily relevant under hypoxic conditions. This is supported by the variable impact of AD-01 observed in MCM-treated 2D and 3D cell cultures, with no significant effect on endothelial cell migration. Nevertheless, HIF-1α regulation of FKBPL is not fully elucidated and whether there are putative sites response elements remains to be determined in the future studies.

In inflammatory conditions, TNF-α stimulates the expression of HIF-1α via NF-κB-dependent transcription [65,66]. Furthermore, we have shown that 24-hour TNF-α exposure also increases FKBPL expression in trophoblast and endothelial cells [67]. FKBPL inhibits NF-κB signalling through its N terminal region, which contains functional and non-functional peptidyl prolyl isomerases [16]. There are other members of the FKBP family, including FKBP51 and FKBP52 that interact with p65 complexes to regulate NF-κB [68]. Although AD-01 is based on non-functional peptidyl prolyl isomerases of FKBPL terminal, it appears this region of FKBPL may play an important role in regulating HIF-1α expression via NF-κB-dependent pathways. However, additional studies are needed to determine the intricate relationships between FKBPL, HIF-1α, TNF-α, and NF-κB.

In a wide range of CVD cases, hypoxia and inflammation often coexist as features of the tissue microenvironment of the dysfunctional endothelium. We showed that under hypoxia, AD-01 regulates proteins and pathways essential for cellular signalling, tissue remodelling, vascular integrity and inflammation. Our proteomics data shows that AD-01 appears to significantly enhance vesicle trafficking, and translocation of nascent secretory and membrane proteins via the activation of the NBAS subunit of NRZ tethering complex and dolichyl-diphosphooligosaccharide-protein glycosyltransferase subunit 2, respectively [69,70]. This finding is in line with previous research showing that FKBPL is a chaperone protein, and an essential component of the Hsp90/estrogen receptor (ER) complex that contributes to the stability and signalling of the ER [71]. AD-01 also enhanced microvascular integrity in conjunction with increasing the expression of the collagen alpha-1(XIX) chain and JCAD, proteins, under hypoxic conditions. Collagen alpha-1(XIX) is a structural component of the basement membrane that forms and maintains the vascular extracellular matrix and has anti-angiogenic function by inhibiting melanoma cell migration [72,73]. Furthermore, AD-01 stabilised the integrity and migratory function of DMOG-treated HMEC-1 cells, likely by increasing the expression of JCAD; a novel component of endothelial cell-cell junctions that plays a key role in pathological angiogenic processes [74]. Indeed, HUVECs exhibited reduced proliferation and migration, increased cell apoptosis, and suppressed Hippo signalling when JCAD was knocked-down [75]. Because AD-01 and FKBPL can modulate the NF-ĸB signalling in response to inflammation, our findings suggest that AD-01 may also regulate NF-ĸB signalling via inhibition of the RIG-I-like receptors and VEGF- pathways [16,76–78]. RIG-I-like receptors stimulate innate immunity and inflammation in endothelial cells by producing type 1 interferon, ICAM, and proinflammatory cytokines, and by inducing the formation of reactive oxygen species. These could impact endothelial function(s) and vascular disorders [79,80].

The main limitation of this study is that we did not validate the role of AD-01 in a hindlimb ischaemia model in vivo, which should be done in future studies. Nevertheless, previous studies have tested AD-01 as a preclinical FKBPL-based peptide candidate therapeutic in various* in vivo* models of cancer [13,81,82] whereas ALM201 (a clinical FKBPL-based peptide candidate therapeutic) was also tested in Phase I clinical trials [14], showing favourable safety profile. Taken together, these findings suggest, for the first time, that FKBPL suppression in hypoxia and upregulation during inflammation can lead to endothelial cell injury likely through HIF-1α overexpression in vascular systems, abrogated by AD-01. Therefore, AD-01 may be potentially applicable as a new treatment of vascular stabilization and anti-inflammatory agent in a wide range of CVD cases. The novel mechanisms of action for AD-01, apart from targeting HIF-1α, involve a number of anti-inflammatory, anti-angiogenic and vascular remodelling proteins in the hypoxic settings, including inhibition of NF-ĸB signalling, and increased expression of collagen alpha 1, and JCAD, proteins. Using this innovative 3D microfluidic endothelial cell model, we demonstrated that complex pathophysiology can be investigated under different treatments and conditions to mimic human tissue damage in CVD, because the microenvironment is highly controllable and dynamic, and it allows real-time observation of cellular interactions and behaviours.

## Electronic supplementary material

Below is the link to the electronic supplementary material.

Supplementary Material 1

## Data Availability

Data can be requested from the corresponding author upon reasonable request.
