# Supplementary material for: The FKBPL-based therapeutic peptide, AD-01, protects the endothelium from hypoxia-induced damage by stabilising hypoxia inducible factor-α and inflammation
Source: J Transl Med. 2025 Mar 11;23:309. doi: 10.1186/s12967-025-06118-w (PMC11895374; doi:10.1186/s12967-025-06118-w)
Supplement: Supplementary file 1 — Supplementary Material 1 [file 12967_2025_6118_MOESM1_ESM.pdf]

FKBPL-based therapeutic peptide, AD-01, protects endothelium from hypoxia-induced damage by stabilising hypoxia inducible factor-α and inflammation

Sahar Ghorbanpour[1,2], Siân Peta Cartland[2], Hao Chen[1], Sanchit Seth [3,4,5] Rupert C. Ecker[3,4,5], Claire Richards[1], Dunja Aksentijevic[6], Matthew P Padula[1], Louise Cole[7], Majid Ebrahimi Warkiani[8], Mary Meltem Kavurma[2], Lana McClements[1#]*

# Corresponding author

1. *School of Life Sciences &* Institute for Biomedical Materials and Devices*, Faculty of Science, University of Technology Sydney, NSW, Australia*
2. Heart Research Institute, The University of Sydney, Sydney, NSW, Australia
3. School of Biomedical Sciences, Faculty of Health, Queensland University of Technology, Brisbane, QLD, Australia.
4. TissueGnostics Australia Pty Ltd, Brisbane, QLD, Australia.
5. Translational Research Institute, 37 Kent Street, Woolloongabba, QLD 4102, Australia
6. Centre for Biochemical Pharmacology, William Harvey Research Institute, Barts and the London School of Medicine and Dentistry, Queen Mary University of London, London, UK.
7. The Australian Institute for Microbiology and Infection (AIMI), Faculty of Science, University of Technology Sydney, NSW, Australia
8. School of Biomedical Engineering, Faculty of Engineering and Information Technology, University of Technology Sydney, NSW, Australia

Keywords: FKBPL, angiogenesis, hypoxia, inflammation, Endothelial dysfunction, Microfluidics

Proteomics sample preparation and Mass spectrometry settings

Briefly, 10 µg of HMEC-1 protein lysates were digested with 100 ng sequencing grade trypsin (Promega, USA) for 18 h at 37°C. The digestion was stopped by adding an equal volume of acetonitrile containing 1 % trifluoroacetic (TFA). To clean, concentrate and enrich the peptide mixtures, STop-And-Go-Extraction tips (STAGE Tips) were used. STAGE Tips were generated by punching double stack 47 mm styrene divinyl benzene-reversed-phase sulfonate (SDB-RPS Empore, 3M) disk by 14-gauge needle and inserted into 100 µL tips. STAGE Tips were activated with 100 µL 100 % acetonitrile and centrifuged at 1000 g for 1 min followed by equilibration step using 100 µL 0.1 % TFA in water and 30 % methanol/1 % TFA with centrifugation at 1000 g for 3 min. Then, each STAGE Tip was loaded with ~ 10 µg digested peptides and washed twice by 100 µL 99 % ethyl acetate/1 % TFA. Afterwards, each STAGE Tip were washed with 100 µL 0.2 % TFA in water and centrifuged at 1000 g for 1 min. For elution of peptides, 100 µL 5 % ammonium hydroxide/80 % acetonitrile was added to each tip and centrifuged at 1000 g for 5 min. The samples were collected in autosampler vials (Thermo Fisher, USA) and dried using SpeedVac vacuum concentrator (Thermo Fisher) at 40 °C for one hour. Finally, dried peptide samples were resuspended in 20 µL 0.1 % formic acid (final concentration 0.5 µg/µL) and analysed by LC-MS/MS as previously described (Roediger et al., 2018). The samples were processed using an Acquity M-class nanoLC system to load 5 µL of sample onto a nanoEase Symmetry C18 trapping column (180 µm × 20 mm) at 15 µL/min rate for 3 min. This was followed by washing of a column equipped with a laser pulled emitter (75 µm ID × 350 mm) filled with SP-120–1.7-ODS-BIO resin (1.7 µm, Osaka Soda Co, Japan) and heated up to 45°C. Peptides were eluted from the column and into the source of a Q-Exactive Plus mass spectrometer (Thermo Fischer): 5–30 % MS buffer (98 % acetonitrile + 0.2 % formic acid) for 90 min, 30-80 % MS buffer for three min, 80 % MS buffer for 2 min, and 80–5 % MS buffer for 3 min. Ionisation of the eluted peptides was performed at 2400 V. A Data Dependent MS/MS (dd-MS^2) experiment was then conducted using 350 – 1500 m/z survey scan (70,000 resolution) with an AGC target of $3e^6$ and 50 milliseconds injection time for peptides with charge states of 2+ or higher. An isolation window of 1.4 m/z, an AGC target of $1e^5$, and a maximum injection time of 100 milliseconds were used to selectively fragment the top 12 peptides in the HCD cell. Using the Orbitrap analyser at 17,500 resolution, fragment masses were measured over a mass range of 120–2000 m/z. Precursor peptide masses were excluded for 30 seconds.

Proteomics data analysis

The setup of software for discovery proteomics experiment was previously described (Chen et al., 2022). The MS/MS data files generated using extracted samples were searched in PEAKS Studio X + (Bioinformatics solution) against the human proteome database (2019). The results of the search were then filtered to include peptides with a $-\log_{10}P$ score that was determined by the False Discovery Rate (FDR) of < 1 %, in which decoy database search matches were < 1% of the total matches. All statistical analyses were performed in R (v4.2.0). The proteomic data were Total Ion Count (TIC)-normalised followed by log2-transformed. A pattern of Missing at Random (MAR) of missing values was found in the data, in which the proteins were detected in some biological replicates but not all in a group. Samples with missing values >85 % were removed. The missing values were imputed using the k-nearest neighbour (KNN) method that is optimal for MAR pattern. Data were filtered for any proteins identified in all biological replicates in at least one group, followed by normalisation using Variance Stabilising Normalisation (VSN) through the DEP package (v1.20.0) (Zhang et al., 2018). Principle component analysis (PCA) was performed using the top 500 variable proteins. Differential enrichment analysis was performed using the limma package (v3.54.2) (Ritchie et al., 2015) for the following comparisons: (1) FKBPL siRNA vs FKBPL siRNA+AD-01, (2) DMOG vs DMOG+AD-01 vs control (3) MCM vs MCM+AD-01 vs control. Significant proteins were defined at a FDR < 0.05. Enriched pathways in AD-01 or stimulus treatment groups were identified by ranking the proteins into descending log2 ratio in individual comparison and performing Gene Set Enrichment Analysis (GSEA) using clusterProfiler (v4.6.2) (Wu et al., 2021; Yu et al., 2012) through all pathways in KEGG database (Kanehisa et al., 2023). Significantly enriched pathways were defined as p-value < 0.05.

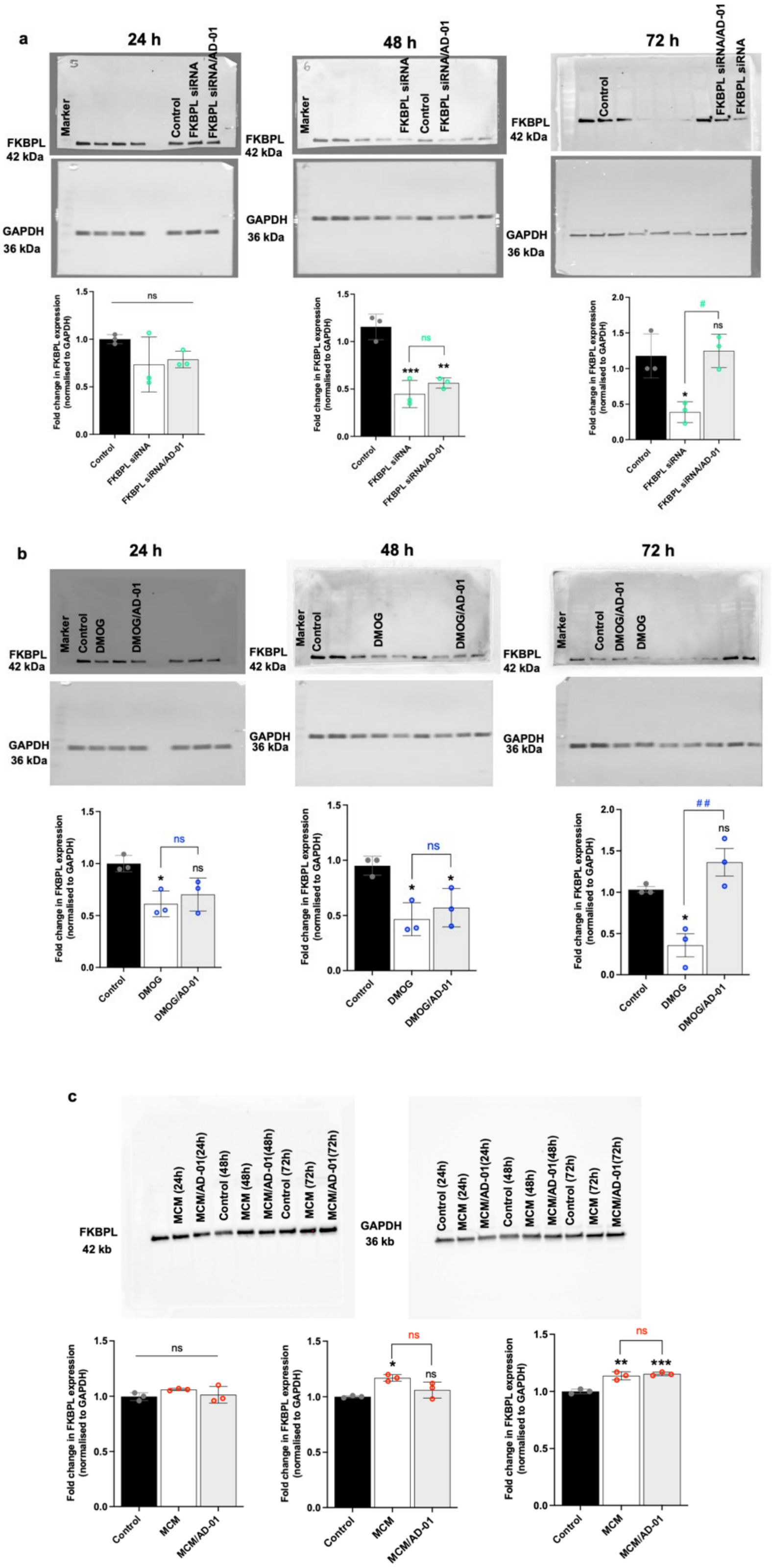

Figure 1: Time-course showing regulation of FKBPL expression in 2D microvascular endothelial cells following FKBPL siRNA, DMOG or MCM, ± AD-01, treatment. The Raw chemiluminescent whole western blot images of HMEC-1 cells protein lysate expression of FKBPL after 24, 48 and 72 h treatments with (**a**) 24 h of treatment with FKBPL siRNA has no effect on FKBPL expression in HMEC-1 cells, while FKBPL siRNA inhibited FKBPL expression by ~ 60% and ~70 % after 48 and 72 h, respectively.(**b**) DMOG treatment reduced FKBPL expression by 40 %, 50 %, and 70 % after 24, 48, and 72 h treatment, respectively. AD-01 restored FKBPL expression only after 72 h of hypoxia. (**c**)

MCM had no effect on FKBPL expression after 24 h of treatment, while significantly increased the expression of FKBPL at 48 and 72 of treatment. SiRNA control, non-targeted siRNA. DMOG, MCM control, untreated cells. FKPBL protein expression was normalized to GAPDH. Results are mean ± SEM, n=3. Data passed Shapiro-Wilk normality test. One-way ANOVA with Tukey post-hoc test, *p<0.05, **p<0.01, ***p<0.001, ****p<0.0001.

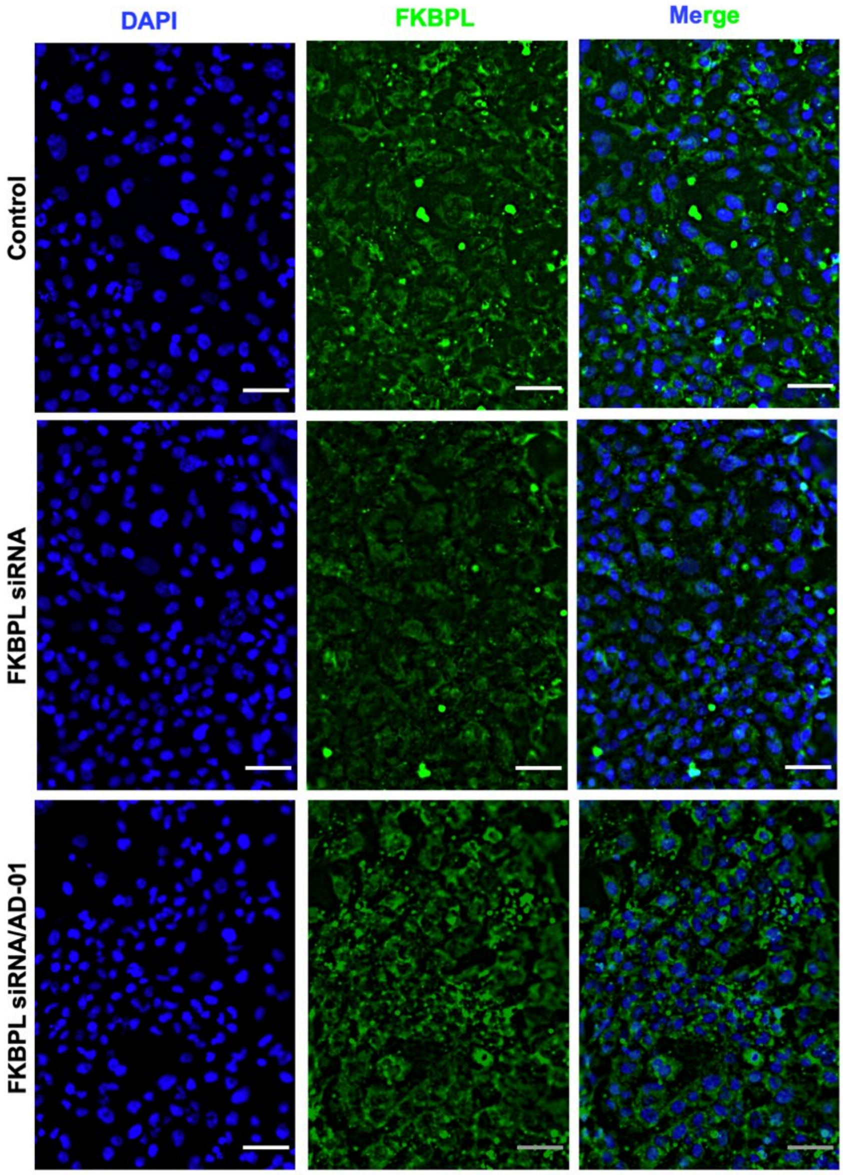

Figure 2: Representative images of FKBPL expression in HMEC-1 cultured in 3D microfluidics chips with collagen. Immunofluorescence images of HMEC-1 cells were taken to quantify the expression of FKBPL, following treatment with 24 µM siRNA ± 100 nM AD-01 for 72 h. Chips were probed for immunofluorescent imaging of FKBPL and DAPI. Scalebars represent 100 µm.

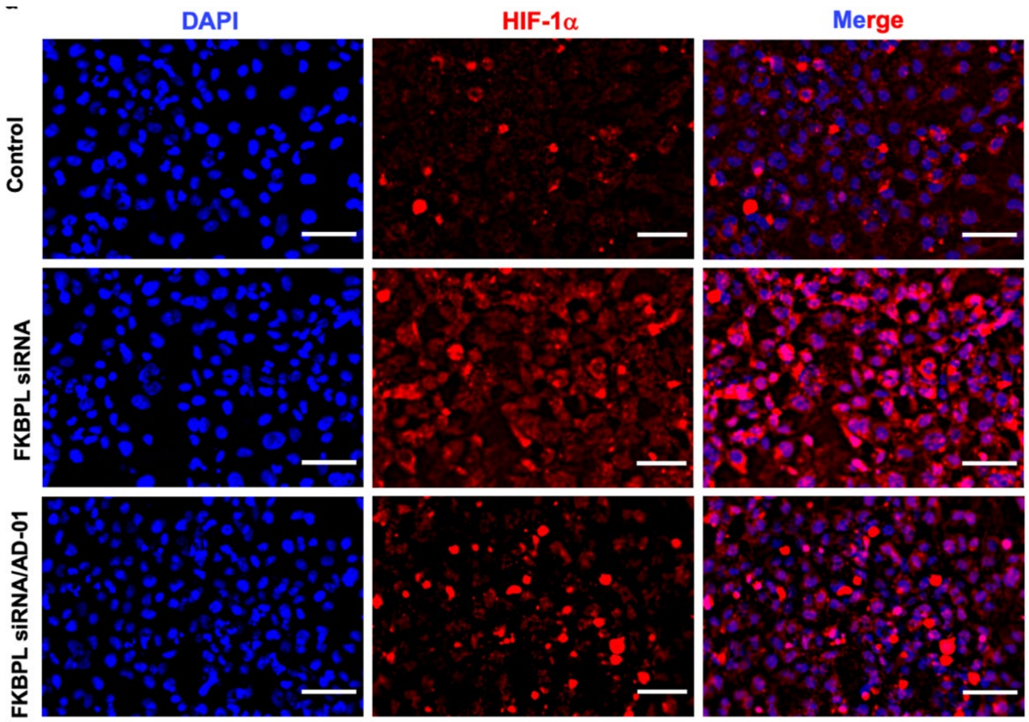

Figure 3: Representative images of HIF-1α protein expression in HMEC-1 following FKBPL knockdown grown in 3D microfluidics chips with collagen. Immunofluorescence images of HMEC-1 cells were taken to quantify the expression of HIF-1α, following treatment with 24 μM siRNA ± 100 nM AD-0 for 72 h. As control for siRNA treatment, non-targeted siRNA was used. Following 72 h of culture, chips were probed for immunofluorescent imaging of HIF-1α and DAPI. Scalebars represent 100 μm.

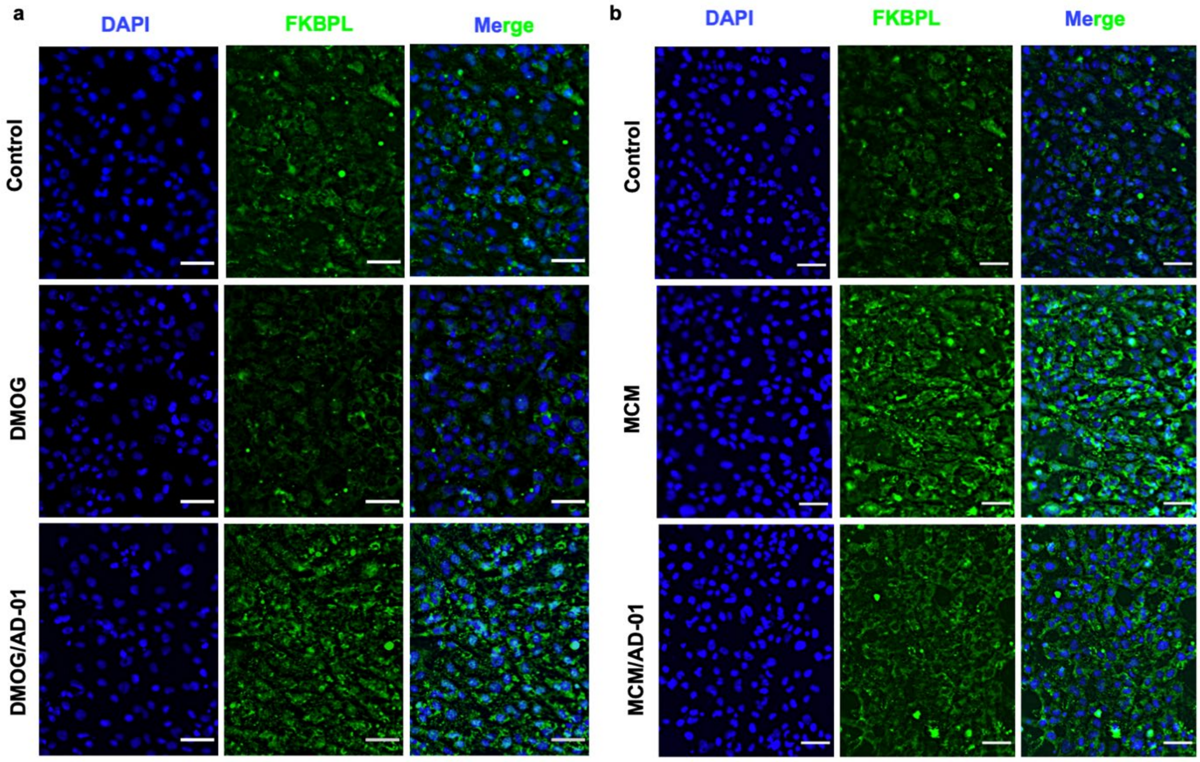

Figure 4: Representative images of FKBPL protein expression in HMEC-1 cultured in 3D microfluidics chips with collagen in the presence of hypoxic stimuli. In 3D-microfluidic setting, collagen matrix (2.5 mg/mL) was added to the central channel and HMEC-1 cells were added to the side channel of microfluidic chips. Cells were treated with 1 mM DMOG and 50 % MCM ± 100 nM AD-01 for 72 h. Following 72 h of culture, chips were probed for immunofluorescent imaging of FKBPL and DAPI. (**a**), (**b**) Representative immunofluorescence images of HMEC-1 cells were taken to quantify the expression of FKBPL, following treatment with DMOG and MCM ± AD-01 respectively. Scalebars represent 100 µm.

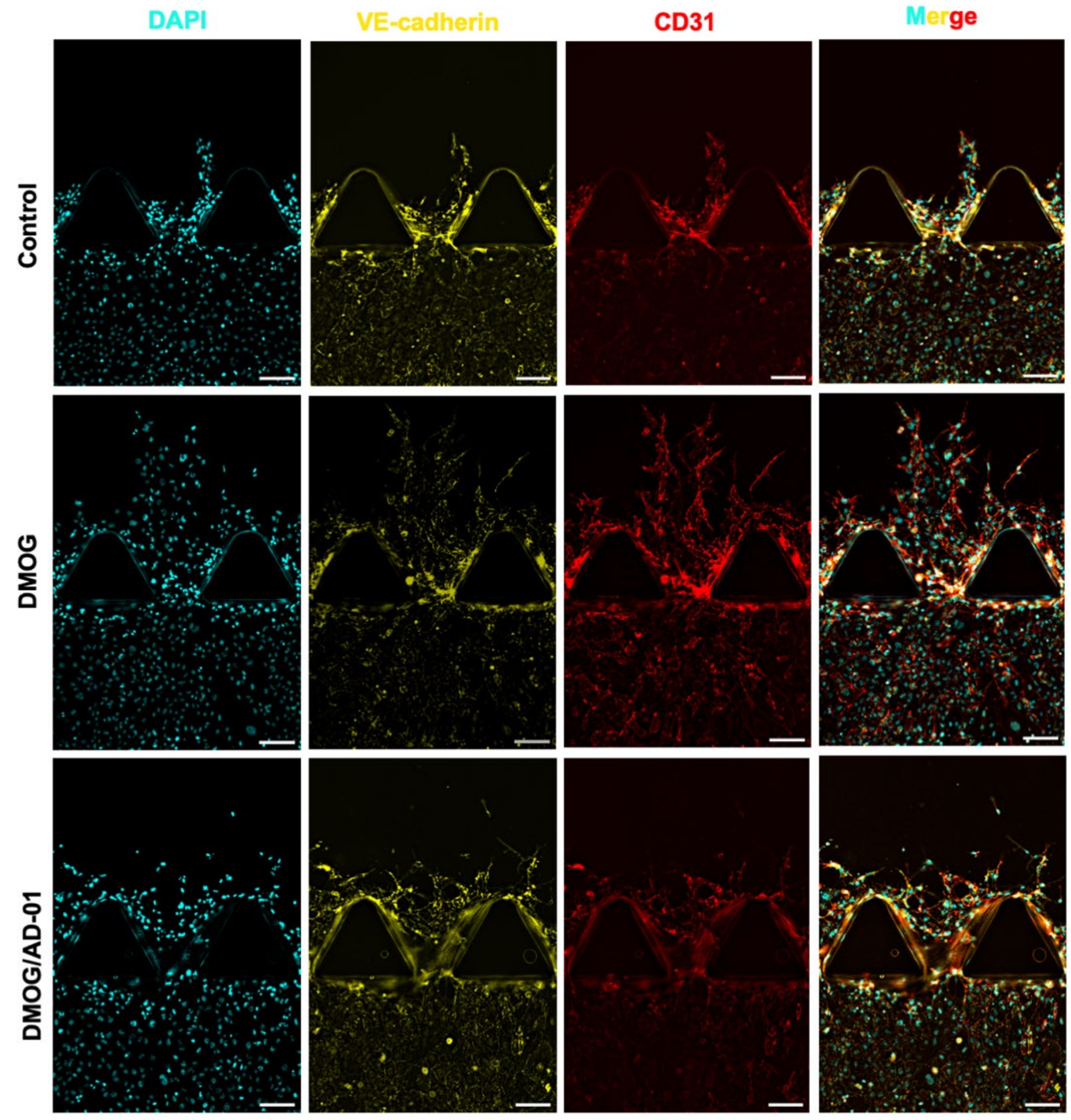

Figure 5: Representative images of VE-cadherin and CD31 protein expression in HMEC-1 cultured in 3D microfluidics chips with collagen in hypoxic conditions. To set up the 3D-microfluidic devices, a collagen matrix (2.5 mg/mL) was added into the central channel and HMEC-1 cells were added to the side channel of microfluidic chips. HMEC-1 cells were treated with 1 mM DMOG ± 100 nM AD-01 for 72 h with untreated cells serving as the control. After 72 h, chips were probed for immunofluorescent imaging of VE-cadherin, CD31 and DAPI. Representative immunofluorescence images of HMEC-1 cells were taken to quantify the expression VE-cadherin, CD31 of following treatment with DMOG ± AD-01, respectively. Scalebars indicate 100 µm.

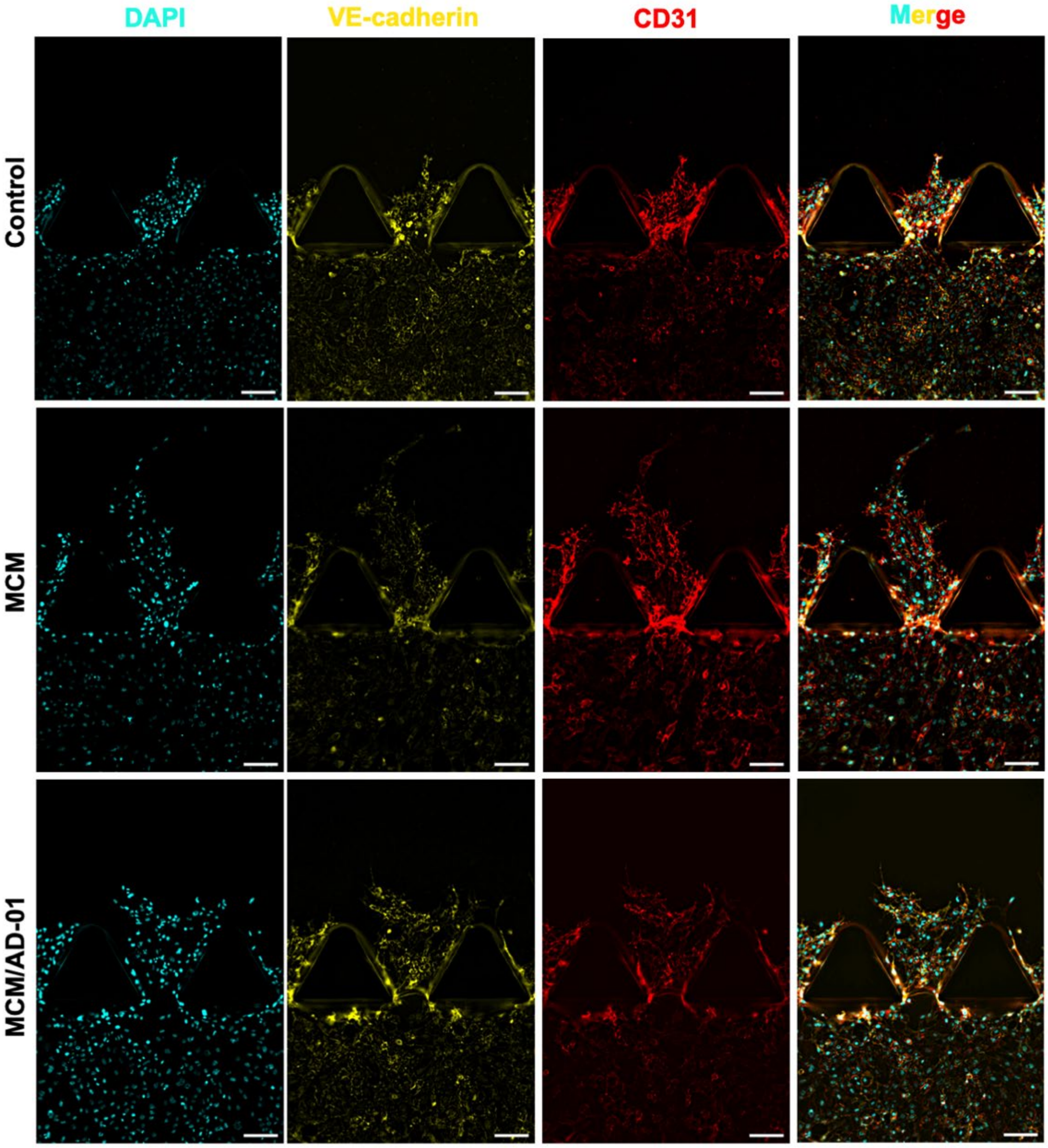

Figure 6: Representative images of VE-cadherin and CD31 protein expression in HMEC-1 cultured in 3D microfluidics chips with collagen in inflammatory conditions. The 3D-microfluidic devices were set up by introducing a collagen matrix (2.5 mg/mL) was added into the central channel and adding HMEC-1 cells to the side channel of microfluidic chips. HMEC-1 cells were treated with 50 % MCM ± 100 nM AD-01 for 72 h with untreated cells as the control. After 72 h, chips were subjected to immunofluorescent imaging of VE-cadherin, CD31 and DAPI. Representative immunofluorescence images of HMEC-1 cells were taken to quantify the expression VE-cadherin, CD31 of following treatment with MCM ± AD-01, respectively. Scalebars indicate 100 µm.

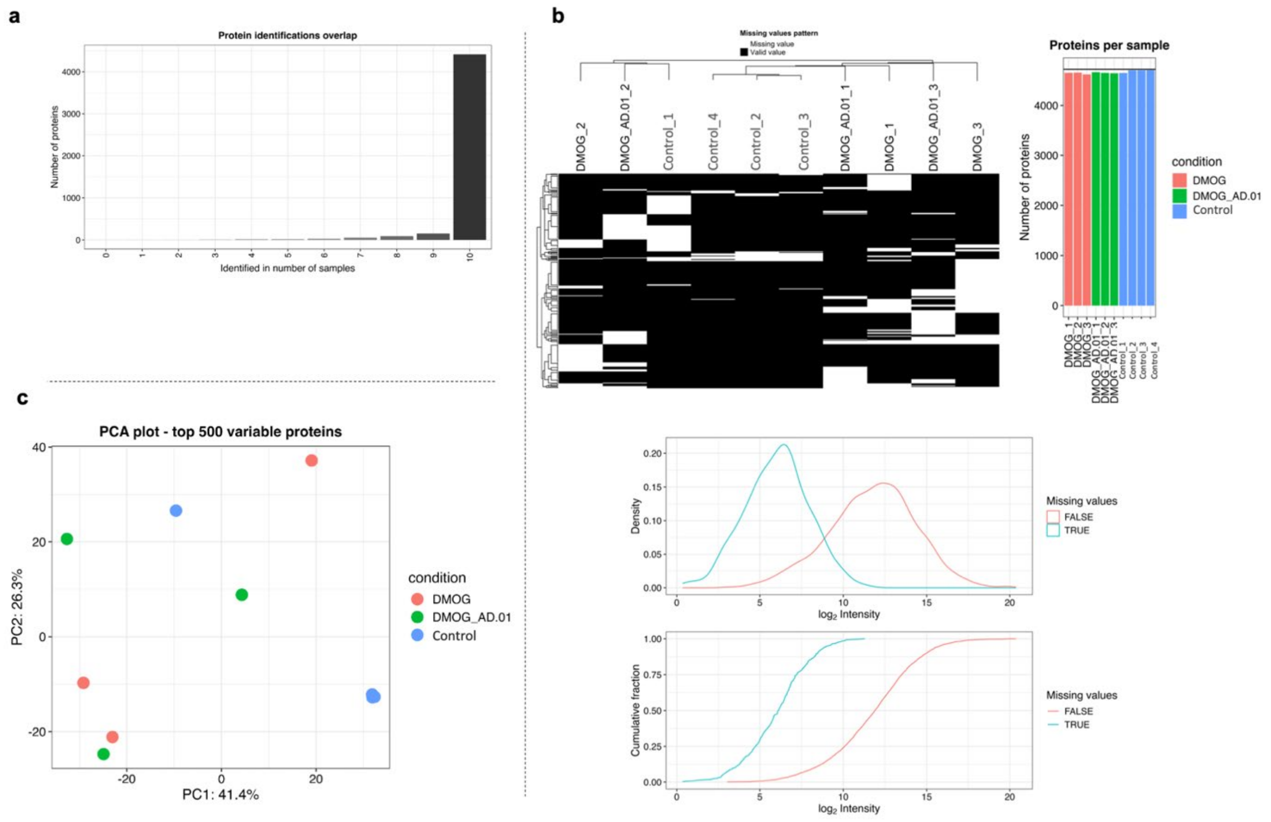

Figure 7: **Pre-processing of proteomics data.** Proteomics results of HMEC-1cells protein lysate after 72 h treatments with 1 mM DMOG ± 100 nM AD-01. (**a**) Investigation of the distribution of missing values across samples. (**b**) Investigation of the pattern of missing values. (**c**) PCA plot of relatively consistent condition-specific clustering of samples.

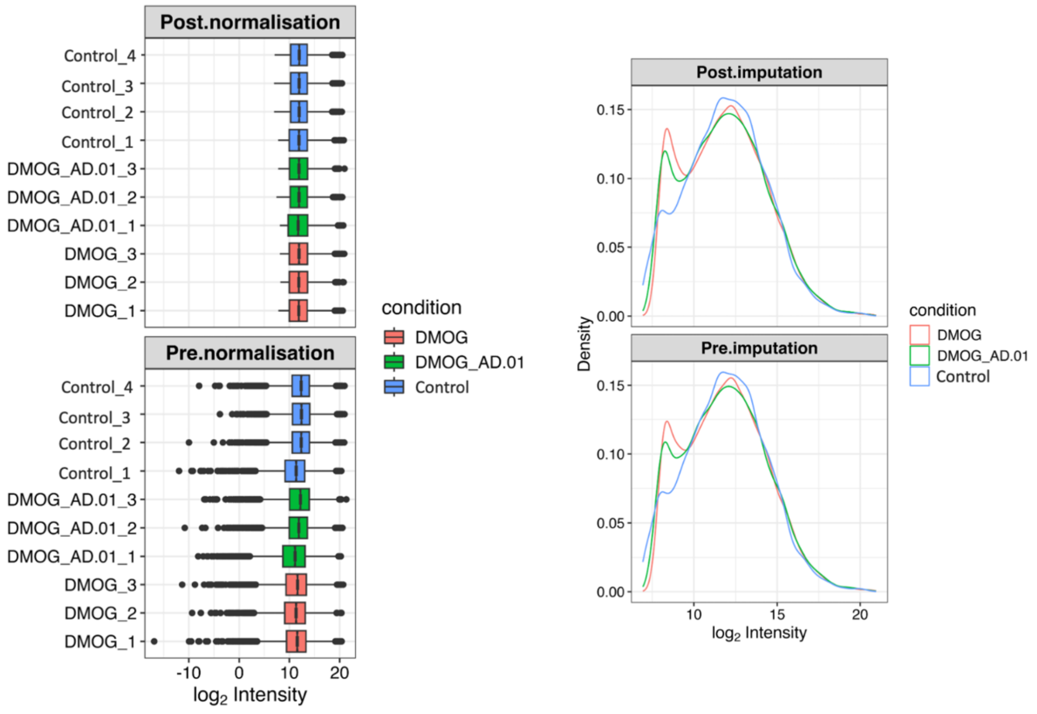

Figure 8: Pre-processing of proteomics data. Comparisons of pre- and post-normalisations and imputations in all analysis cascades of DMOG, DMOG + AD-01 and control.

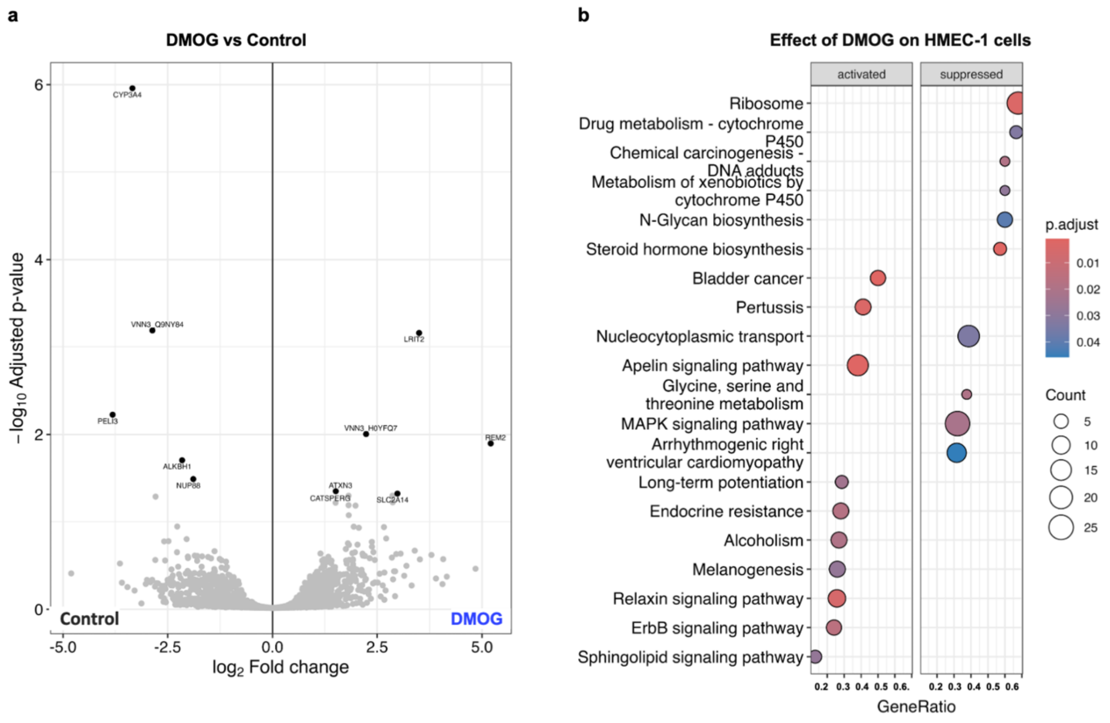

Figure 9: Proteome investigation of the effect of DMOG on HMEC-1 cells. (**a**) Differential enrichment analysis comparing HMEC-1 cells treated with DMOG and control. (**b**) Comparison between top 20 pathways enriched in HMEC-1 cells treated with DMOG.

Table 1: Downstream protein targets of DMOG in HMEC cells. A description of down- and up-regulated proteins identified when DMOG and Control proteomic profiles were compared.

Protein group(s)	Gene ID	Protein description	p-Value	Log FC
ALKBH1	H0YJF3	AlkB homolog 1, histone H2A dioxygenase	0.0197	-2.16
CYP3A4	A0A494C0W7	Cytochrome P450 family 3 subfamily A member 4	1.1e-06	-3.34
NUP88	J3KMX1	Nucleoporin 88	0.0324	-1.89
PELI3	Q8N2H9	E3 ubiquitin-protein ligase pellino homolog 3	0.00596	-3.81
CATSPERG	B8ZZI7	Cation channel sperm associated auxiliary subunit gamma	0.0447	1.51
LRIT2	A6NDA9	Leucine-rich repeat, immunoglobulin-like domain and transmembrane domain-containing protein 2	0.000694	3.5
VNN3_H0YFQ7	H0YFQ7	Vanin 3	0.0099	2.24
REM2	Q8IYK8	GTP-binding protein REM 2	0.0127	5.21
SLC2A14	Q8TDB8	Solute carrier family 2, facilitated glucose transporter member 14	0.0476	2.98

Table 2 Downstream protein targets of AD-01 on DMOG-treated HMEC cells. A description of down- and up-regulated proteins identified when DMOG and DMOG/AD-01 proteomic profiles were compared.

Protein group(s)	Gene ID	Protein description	p-Value	Log FC
KIR3DL3	A0A0B4J1R5	Killer cell immunoglobulin-like receptor 3DL3	0.033	1.18
MYO1A	Q9UBC5	Unconventional myosin-Ia	0.00873	-1.15
NAP1L1	B7Z9C2	Nucleosome assembly protein 1 like 1	0.000614	-1.76
RGS7	P49802	Regulator of G-protein signaling 7	0.0343	-2.26
SEPTIN10_B5ME97	B5ME97	Septin 10	1.7e-05	-1.89
TJP3	O95049	Tight junction protein-3	0.0407	-1.21
WDR33	Q9C0J8	pre-mRNA 3' end processing protein WDR33	0.00872	-2.2
XPOT	O43592	Exportin-T	0.0416	-1.02
PLEKHG2	Q9H7P9	Pleckstrin homology domain-containing family G member 2	0.00687	-2.13
ACSM2A	E7EPS5	Acyl-CoA synthetase medium chain family member 2A	0.0149	2.18

ALKBH1	H0YJF3	AlkB homolog 1, histone H2A dioxygenase	0.0273	1.33
COL19A1	Q14993	Collagen alpha-1(XIX) chain	0.000573	3.03
COPS7B	J3QT95	COP9 signalosome subunit 7B	0.0104	1.74
CYP2D6	A0A0G2JLK7	Cytochrome P450 2D6	0.000547	1.28
DDX11	Q96FC9	ATP-dependent DNA helicase DDX11	0.0119	1.84
ECT2	Q9H8V3	Protein ECT2	0.0378	1.29
IRAG1	Q9Y6F6	Inositol 1,4,5-triphosphate receptor associated 1	0.0451	1.68
JCAD	Q9P266	Junctional cadherin 5-associated protein	0.00821	1.27
KCNA3	P22001	Potassium voltage-gated channel subfamily A member 3	0.00481	1.53
LMNTD1	Q8N9Z9	Lamin tail domain-containing protein 1	0.0416	1.73
LOC101059915	A0A1B0GWI6	Uncharacterized protein	0.0456	1.07
NBAS	H0Y5G7	NBAS subunit of NRZ tethering complex	0.0368	1.42
PIDD1	Q9HB75	p53-induced death domain-containing protein 1	0.00857	1.88
PRKG1	A0A2R8Y507	Protein kinase cGMP-dependent 1	0.0135	2.13
Protein_kinase_domain-containing_protein_fragment	M0QX08	Protein kinase domaincontaining protein	0.0397	2.32
PRR15	Q8IV56	Proline-rich protein 15	0.0308	1.57
PSPC1	Q8WXF1	Paraspeckle component 1	0.0399	3.28
RAPGEF3	O95398	Rap guanine nucleotide exchange factor 3	0.0417	1.24
RGMA	A0A0A0MTQ4	Repulsive guidance molecule A	0.00611	2.45
RPN2	P04844	Ribophorin 2	0.0292	1.69
SFTPD	P35247	Pulmonary surfactant-associated protein D	0.0469	1.47
SIX6OS1	Q8N1H7	Protein SIX6OS1	0.0147	2.92
SLC2A11	F8WB79	Solute carrier family 2 member 1	0.0133	3.72
SLFN11	Q7Z7L1	Schlafen family member 11	0.0483	1.37
TRPC7	Q70T25	Transient receptor potential cation channel subfamily C member 7	2.08e-06	2.54

RPN2	P04844	Dolichyl-diphosphooligosaccharide--protein glycosyltransferase subunit 2	0.0292	1.69
SFTPD	P35247	Pulmonary surfactant-associated protein D	0.0469	1.47
SIX6OS1	Q8N1H7	Protein SIX6OS1	0.0147	2.92
SLC2A11	F8WB79	Solute carrier family 2 member 11	0.0133	3.72
SLFN11	Q7Z7L1	Schlafen family member 11	0.0483	1.37
TRPC7	Q70T25	Transient receptor potential cation channel subfamily C member 7	2.08e-06	2.54
TYRP1	P17643	5,6-dihydroxyindole-2-carboxylic acid oxidase	0.0372	1.38
UGGT2	Q9NYU1	UDP-glucose:glycoprotein glucosyltransferase 2	0.00759	2.31
ZSCAN5B	A6NJL1	Zinc finger and SCAN domain-containing protein 5B	0.00772	1.82
